# Analysis of the Equilibrium Phase in Immune-Controlled Tumors Provides Hints for Designing Better Strategies for Cancer Treatment

**DOI:** 10.3389/fonc.2022.878827

**Published:** 2022-06-27

**Authors:** Kevin Atsou, Sokchea Khou, Fabienne Anjuère, Véronique M. Braud, Thierry Goudon

**Affiliations:** ^1^ Université Côte d’Azur, Inria, CNRS, LJAD, Nice, France; ^2^ Université Côte d’Azur, CNRS, Institut de Pharmacologie Moléculaire et Cellulaire UMR 7275, Valbonne, France

**Keywords:** cancer, mathematical oncology, equilibrium phase, immunotherapy, drug response

## Abstract

When it comes to improving cancer therapies, one challenge is to identify key biological parameters that prevent immune escape and maintain an equilibrium state characterized by a stable subclinical tumor mass, controlled by the immune cells. Based on a space and size structured partial differential equation model, we developed numerical methods that allow us to predict the shape of the equilibrium at low cost, without running simulations of the initial-boundary value problem. In turn, the computation of the equilibrium state allowed us to apply global sensitivity analysis methods that assess which and how parameters influence the residual tumor mass. This analysis reveals that the elimination rate of tumor cells by immune cells far exceeds the influence of the other parameters on the equilibrium size of the tumor. Moreover, combining parameters that sustain and strengthen the antitumor immune response also proves more efficient at maintaining the tumor in a long-lasting equilibrium state. Applied to the biological parameters that define each type of cancer, such numerical investigations can provide hints for the design and optimization of cancer treatments.

**Graphical Abstract:**
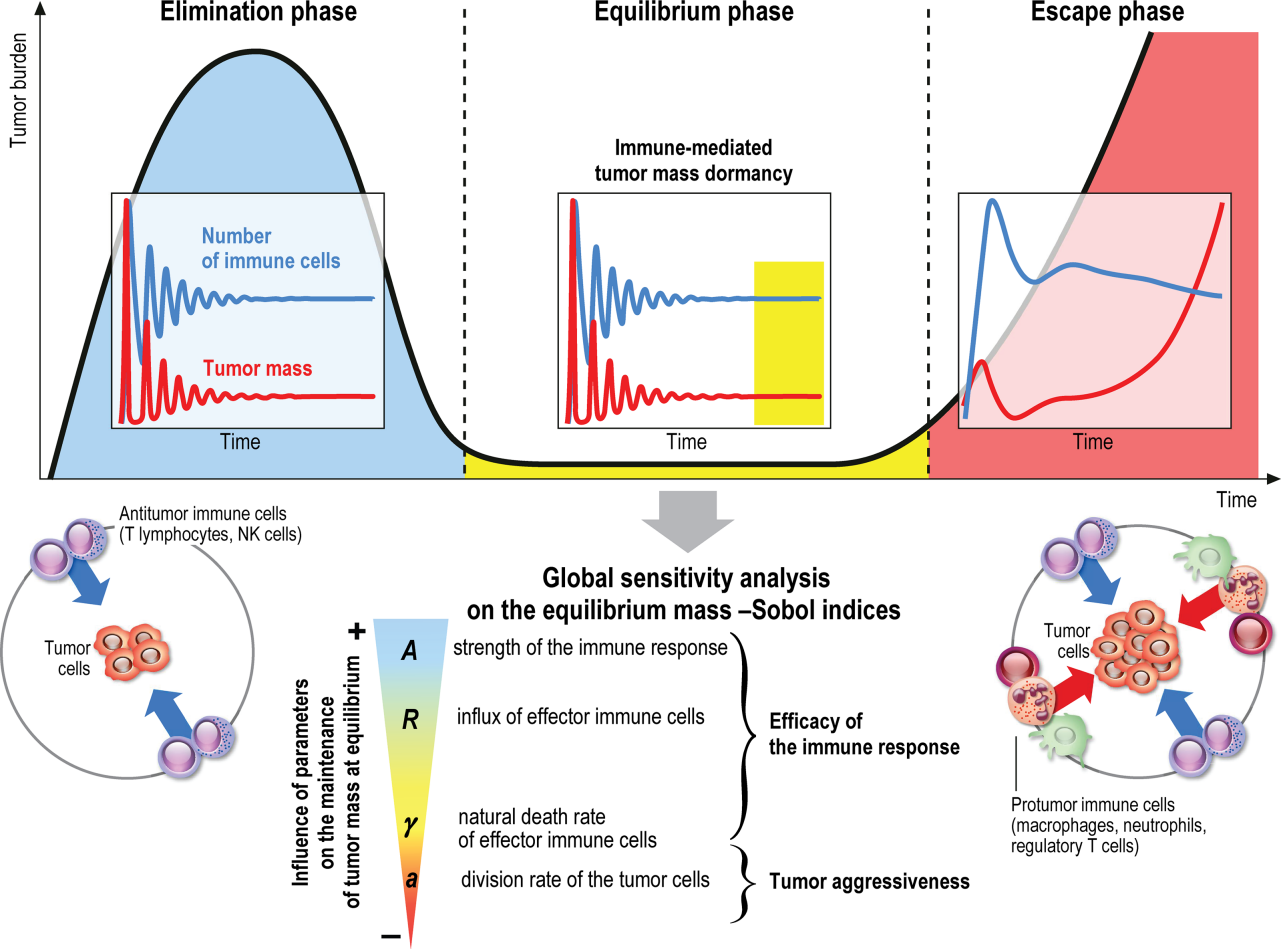


## 1 Introduction

The immune system plays a major role in the control of tumor growth. This has led to the concept of immune surveillance and cancer immunoediting composed of three phases (1–3): the elimination, when tumors are rapidly eradicated by the immune system, the equilibrium, a latency period when tumors can survive but remain on a controlled state, and the escape, the final outgrowth of tumors that have outstripped immunological restraints. In this later phase, immune suppression is prevailing and immune cells are also subverted to promote tumor growth. Numerous cancer immunotherapy strategies have been designed and assessed to counteract immune suppression and restore effective and durable elimination of tumors (4–8). They show improved efficacy over conventional anticancer treatments but only a minority of patients respond. The challenge to face now is to identify key biological parameters which will convert a fatal outcome into a chronic, manageable state, the durable maintenance of cancer in a viable equilibrium phase controlled by immunity. Reaching such immune-mediated tumor mass dormancy is indeed the first key step for successful control of tumor growth and a goal for immunotherapy (9). The equilibrium state is however difficult to apprehend experimentally because the tumor mass at equilibrium is below detectable limits (3). Mathematical modeling of the tumor-immune system interactions offers useful information about the features of the equilibrium phase during primary tumor development, and such tools could be used to guide the design of optimal anticancer therapies (10–13).

We previously (10) introduced a specific multiscale mathematical model based on partial differential equations (PDE), intended to describe the earliest stages of tumor-immune system interactions. We conjecture that the space heterogeneities of the distribution of active and resting immune cells, which are subjected to several interaction mechanisms with the tumor cells, plays a critical role in the efficiency of the immune response, and the ability in reaching the equilibrium phase. This, in turn, motivates the appeal to PDEs descriptions and can complete the already established modeling based on ordinary differential systems, on which there exists a wide literature, see for instance (11, 14–19) Extension to the PDE framework has permitted to bring out the role of space organisation (20–23). The reader can find further details and references about the mathematical modeling of tumor-immune system interactions, based on different viewpoints and addressing several issues of the efficacy of the immune response, in the reviews (24–29). The original model developed in (10) thus accounts for both the growth of the tumor, by natural cell growth and cell divisions, and the displacement of the immune cells towards the tumor, by means of activation processes and chemotaxis effects. The most notable finding from (10) was that an equilibrium state, with residual tumor and active immune cells, can be observed. Moreover, mathematical analysis provides a basis for the explanation of the formation of the equilibrium. How the biological parameters shape this equilibrium is the main question investigated in the present article. Indeed, the equilibrium can be mathematically interpreted by means of an eigenproblem coupled to a stationary diffusion equation with constraint. This observation permits us to develop an efficient numerical strategy to determine *a priori* the shape of the equilibrium — namely, the size distribution of the tumor cells and the residual tumor mass — for a given set of biological tumor and immune cell parameters. Consequently, the equilibrium state can be computed at low numerical cost since we can avoid the resolution of the evolution problem on a long time range. The use of this simple and fast algorithm allows us to address the question of the sensitivity of the residual mass to the parameters and to discuss the impact of treatments. This information can be decisive to design clinical studies and choose therapeutic strategies that will revert to an equilibrium phase. Our work therefore provides hints for cancer treatment management.

### 1.1 Quick Guide to Equations: A Coupled PDE Model for Tumor-Immune System Interactions

**Figure 1:**
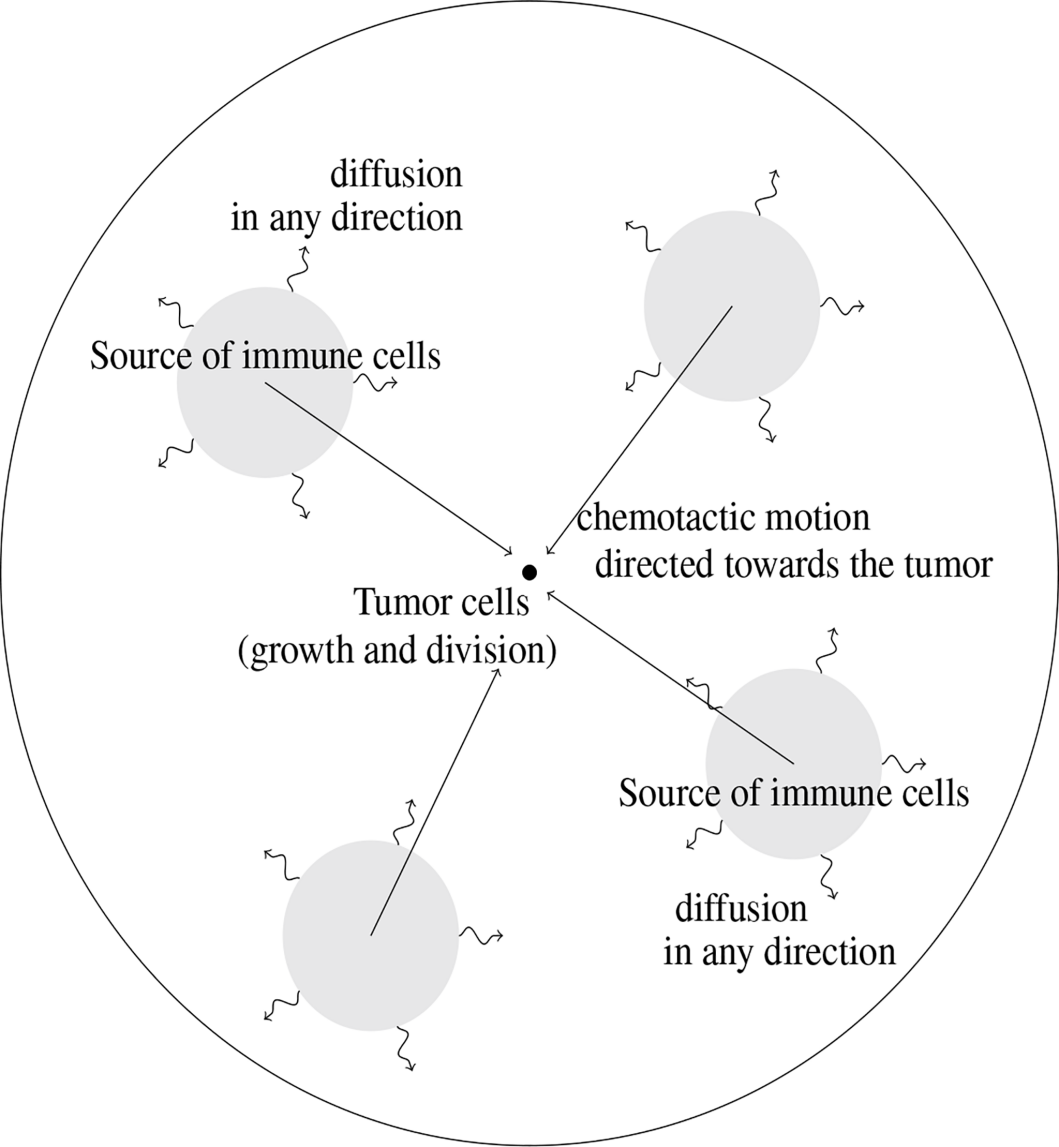
Schematic view of the geometry of the mathematical model. The tumor cells are located at the center of the domain where they are subjected to growth and division mechanisms. Immune cells are activated from baths of resting cells; their motion is driven by diffusion combined to a convection field, due to chemotactic mechanisms and directed towards the tumor.

The modeling approach imposes to select a few phenomena, considered as the leading effects for the situation under consideration; other effects are just roughly described by tuning some parameters or are simply disregarded. Choices for designing the mathematical model are also dictated by the difficulty in attributing numerical values to the parameters of the equations, due to a lack of experimental measurements: the poor knowledge of driving quantities leads to keep a description as simple as possible, with a reduced number of unknown parameters. The principles of the modeling adopted in (10), summarized by **Figure 1**, led to couple an evolution equation for the size-distribution of the tumor cells, and a convection-diffusion equation for the activated immune cells. The two-way coupling arises from the death term induced by the action of the immune cells on the tumor cells, and by the activation and the attraction of immune cells towards the tumor, which are determined by the total mass of the tumor. The model is intended to describe the earliest stages of the tumor formation, when the size of the tumor is relatively small. The tumor is located at the center of a domain Ω (there is no displacement of the tumor). The model distinguishes two distinct and independent length scales: the size of the tumor cells, described by the variable *z*≥0 , is considered as “infinitely small” compared to the scale of displacement of the immune cells, described by the space variable *x* ∈ *Ω* .

The unknowns are

- The size density of tumor cells ( *t*, *z* )↦*n*( *t*, *z* ) so that the integral $\int_{a}^{b}z\;n\left(t,\;z\right)\text{ d}z$ gives the volume of the tumor occupied at time *t* by cells having their size *z* in the interval (*a, b*);
- The concentration of activated immune cells which are fighting against the tumor ( *t*, *x* )↦*c*( *t*, *x* ) ;
- The concentration of chemical signal that attracts the immune cells towards the tumor microenvironment ( *t*, *x* )↦*ϕ*( *t*, *x* ).

**Figure 2:**
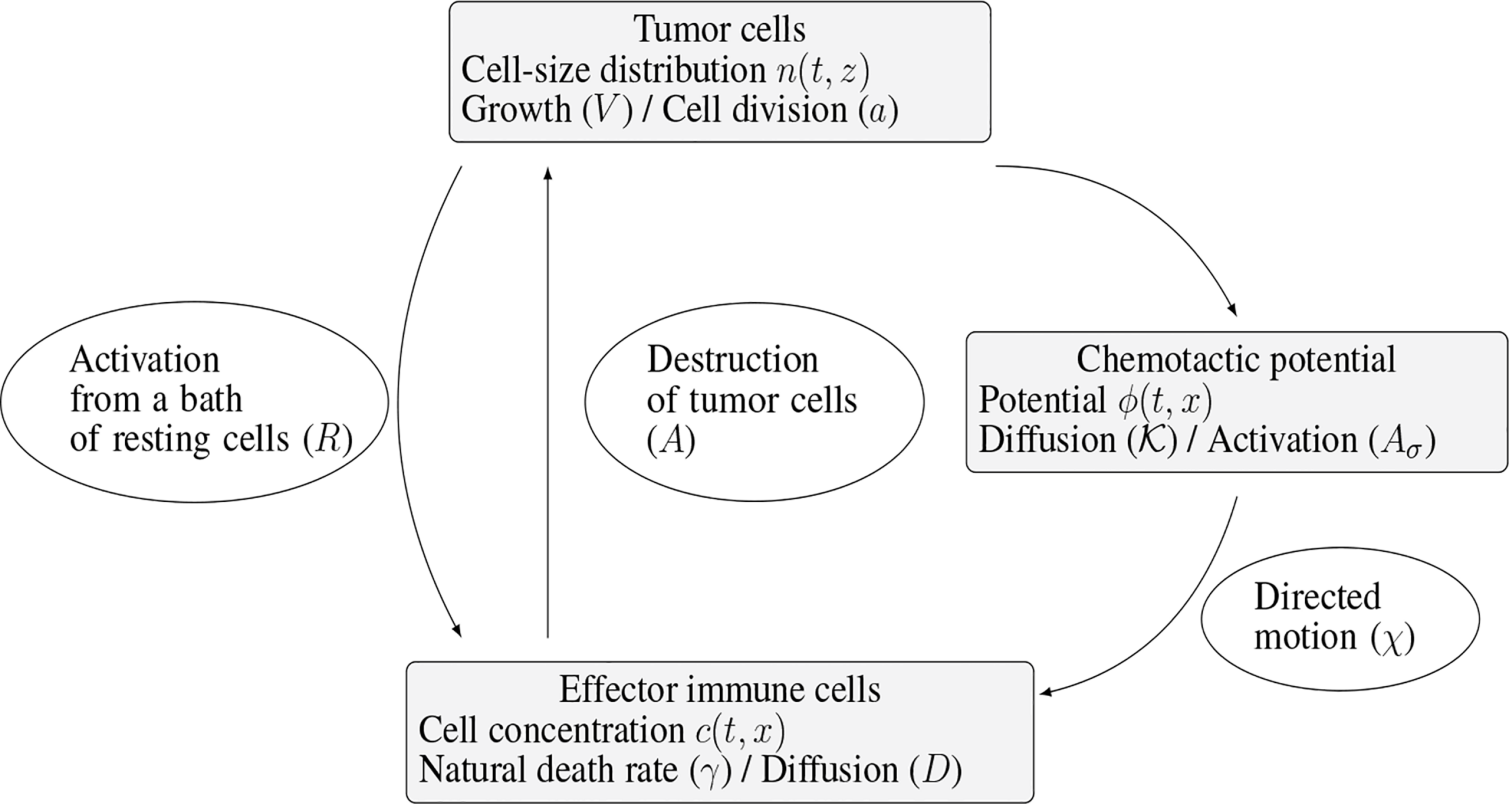
Schematic view of the interaction mechanisms described by the system (1a)-(1e).

The specific biological assumptions made to construct the model are fully described in (10). **Figure 2** offers an overview of the interaction mechanisms embodied in the equations and of the role of the parameters of the model.

Immune cells, once activated from a bath of resting cells, are subjected to natural diffusion and to a chemotactic drift, induced by the presence of the tumor. The strength of this drift, as well as the activation of immune cells, directly depends on the total mass of the tumor, proportional to the quantity


$$\mu_{1}\left(t\right)=\int_{0}^{\infty }zn\left(t,z\right)\text{ d}z.$$

The immune system-tumor competition is described by the following system of PDEs


(1a) $$\partial_{t}n+\partial_{z}\left(Vn\right)=Q\left(n\right)-m\left(n,c\right),$$

(1b) $$\partial_{t}c+\nabla_{x}\cdot \left(c\chi \nabla_{x}\phi -D\nabla_{x}c\right)=\mu_{1}R-\gamma c,$$

(1c) $$-K{\text{Δ}}_{x}\phi =\mu_{1}\left(\sigma \left(x\right)-\frac{1}{\left|\Omega \right|}\int_{\text{Ω}}\sigma \left(y\right)\text{ d}y\right),$$

(1d) $$n\left(t,0\right)=0,\;c{|}_{\partial \text{Ω}}=0,\;K\nabla_{x}\phi \cdot \nu {|}_{\partial \text{Ω}}=0,$$

(1e) $$n\left(t=0,z\right)=n_{0}\left(z\right),\;c\left(t=0,x\right)=c_{0}\left(x\right).$$

The features of the growth-division dynamics for the tumor cells (1a) are embodied into the (possibly size-dependent) growth rate *z*↦*V*( *z* )≥0 and the cell division operator *Q*(*n*). We refer the reader to (30–37) for further details on this evolution equation (with *m* (*n*, *c*) =0) for cell growth and division, and its application to cancer modeling. What is crucial for modeling purposes is the principle that cell-division does not change the total mass: the operator *Q* satisfies $\int_{0}^{\infty }zQ\left(n\right)\text{ d}z=0$ . However, the total number of cells in the tumor increases since $\int_{0}^{\infty }Q\left(n\right)\text{ d}z\geq 0$ (we refer the reader to (10) and Appendix A for further details). In what follows, we restrict to the mere symmetric binary division operator


(2) Q(n)(t,z)=a(z)(4n(t,2z)−n(t,z)),

with *z*↦*a*(*z* )≥0 the division rate. It simply describes the situation where cells are cut into two cells having half the size of the original cell. Further relevant examples of division operators can be found in (32) (see Appendix A). The specific case where the division rate *a* in (2) is a positive constant makes the model simpler, and is often used. It is however likely relevant to incorporate more complex behaviors through the size-dependence; for instance divisions can be prohibited below a certain size threshold. Similarly, it can be convenient to assume that the growth rate *V* is a positive constant, but more intricate laws can take into account some important phenomena. For instance, logistic or Gompertz law can incorporate size limitation effects, and roughly describe difficulties in accessing nutrients or necrotic effects (38–40); a detailed study of growth laws can be found in (41). As mentioned above, though, using such complex laws, also raises the issue of determining more parameters. The boundary condition for *n* in (1d) means that no tumor cells are created with size 0.

Despite the fact that there exists several types of immune cells – at least T-cells and NK cells – fighting against the tumor, they are all described here through the single concentration *c*. It also means that coefficients of the equation – the death rate *γ*>0, the chemotactic strength *χ*>0 , and the diffusion coefficient *D* – correspond to an averaged behavior of all these cells. By the way, working with a constant diffusion coefficient *D* > 0 is again a simplification, neglecting the architecture of the tumor environment, which might induce directional effects. The effector immune cells that effectively fight against the tumor, are activated from a “reservoir” of resting cells, described in the right hand side of (1b) by ( *t*,*x* )↦*R*( *t*,*x* ) . This given function, possibly time and space dependent, stands for the space distribution of the influx rate of activated effector immune cells. It takes into account the sources of resting immune cells that can be activated in the tumor microenvironment or in the draining lymph nodes into cells fighting the tumor. At early stages of tumor growth, the rate of the activation process is supposed to be directly proportional to the tumor mass μ_1_. Again, more complex activation law, for instance based on Michaelis-Menten kinetics can incorporate relevant limitation mechanisms. The Dirichlet boundary condition for *c* in (1d) means that the immune cells far from the tumor are non-activated. Immune cells are directed towards the tumor by a chemo-attractive potential *ϕ*, induced by the presence of the tumor cells. Through (1c), the strength of the signal is proportional to the total mass of the tumor, and it is shaped by a form function *x*↦*σ*( *x* ) which will be a function peaked at the tumor location. The potential is thus defined by the diffusion equation (1c), that involves a positive coefficient *K*>0 (that could be matrix valued), and the Neumann boundary condition in (1d), where *v* stands for the unit outward normal vector on ∂*Ω*. Finally, the activated immune cells are able to destroy tumor cells, as described by the death term in (1a)


(3) $$m\left(c,n\right)\left(t,z\right)=\underset{:=\mu_{c}\left(t\right)}{\underbrace{\int_{\Omega }\delta \left(y\right)c\left(t,y\right)\text{d}y}}\times n\left(t,z\right),$$

where *δ*≥0 is another form function, also peaked in the vicinity of the tumor. For the numerical experiments, we shall work with the Gaussian profiles


(4) $$\begin{array}{cc} \delta \left(x\right)=\frac{A}{\theta \sqrt{2\pi }}\text{exp }\left(-\frac{|x{|}^{2}}{2\theta^{2}}\right), & \sigma \left(x\right)=\frac{A_{\sigma }}{\theta_{\sigma }\sqrt{2\pi }}\text{exp }\left(-\frac{|x{|}^{2}}{2\theta_{\sigma }^{2}}\right), \end{array}$$

where the positive parameters *A*,*A*_*σ*_ and *θ*,*θ*_*σ*_ can be used to tune the amplitude and spreading of these functions, and thus the strength and radius of influence of the related phenomena. We refer the reader to (10) for further details and comments about the model. Note that this model neglects the possible additional protumoral effects that can take place and are crucial to swing to the escape phase. Such protumor effects can have different forms: they can directly enhance the tumor growth, and make antitumor immune cells exhausted, a state where they are hyporesponsive and cannot kill the tumor, see (42) on these issues. Remarkably, the model (1a)-(1e) is able to reproduce equilibrium phases where the tumor growth is controlled by the immune response.

## 2 Materials and Methods

### 2.1 Development of Numerical Methods Predicting Parameters of the Equilibrium in Immune-Controlled Tumors

According to (2, 3, 9), the equilibrium phase corresponds to a long-lasting period of immune-mediated latency, also known as tumor mass dormancy, prior to the emergence of clinically detectable malignant disease, with a residual tumor which has not be fully destroyed by the immune system, maintained under the control of immunity. The simulations of the initial-boundary value problem (1a)-(1e) performed in (10) revealed that such a behavior can be reproduced by the model. Here, we wish to study the features of the equilibrium phase in immune-controlled tumors and, in particular, we want to predict, for given biological parameters (see Section 2.2 below), the total mass of the residual tumor and its size distribution. To this end, we developed specific numerical procedures based on the mathematical interpretation of the equilibrium.

#### 2.1.1 Equilibrium States

The definition of the equilibrium relies on the following arguments. When disregarding the immune response, the cell-division equation


(5) $$\partial_{t}n+\partial_{z}\left(Vn\right)=Q\left(n\right).$$

admits a positive eigenstate, which drives the large time behavior of the solution. To be more specific, there exists *λ*>0 and a non negative function $z\geq 0\mapsto \bar{N}\left(z\right)$ satisfying


(6) $$\{\begin{array}{l} \partial_{z}\left(V\bar{N}\right)-Q\left(\bar{N}\right)+\lambda \bar{N}=0\text{ for }z\geq 0\\ \begin{array}{ccc} \bar{N}\left(0\right)=0, & \bar{N}\left(z\right)>0\text{ for z}>\;0, & \int_{0}^{+\infty }\bar{N}\left(z\right)\text{ d}z=1. \end{array} \end{array}$$

The existence-uniqueness of the eigenpair $\left(\lambda ,\bar{N}\right)$ can be found in (32, 34). Furthermore, when the tumor does not interact with the immune system, the large time behavior is precisely driven by the eigenpair: the solution of (5) behaves like


$$n\left(t,z\right)\underset{t\to \infty }{∼}\mu_{0}e^{\lambda t}\bar{N}\left(z\right)$$

where *μ*_0_ >0 is a constant determined by the initial condition, see (33, 34). Consequently, in the immune-free case, the tumor population grows exponentially fast, with a rate *λ*>0 , and, as time becomes large, its size repartition obeys a certain profile $\bar{N}$ . In the specific case where *V* is constant and *Q* is the binary division operator (2), with a constant division rate *a*, we simply have *λ*=*a* and the profile $\bar{N}$ is explicitly known (43, 44). However, for general growth rates and division kernels the solution should be determined by numerical approximations; we are going to detail a numerical procedure to effectively compute the pair $\left(\lambda ,\bar{N}\right)$ .

Coming back to the coupled model (1a)-(1e), we infer that the equilibrium phase corresponds to the situation where the death rate – the integral of the immune cells concentration with weight δ, denoted as ${\bar{\mu }}_{c}$ in (3) – precisely counterbalances the natural exponential growth of the tumor cell population. In other words, at equilibrium we expect that

• The size distribution of tumor cells is proportional to the eigenstate $\mu_{0}\bar{N}\left(z\right)$ . The proportionnality factor is related to the total mass by the relation $\mu_{1}=\mu_{0}\int_{0}^{\infty }z\bar{N}\left(z\right)\text{ d}z$ .

• The concentration of immune cells is defined by the stationary equation


(7) $$\begin{array}{cc} \gamma C-\nabla_{x}\cdot \left(D\nabla_{x}C\right)+\mu_{1}\nabla_{x}\cdot \left(\chi C\nabla_{x}\text{Φ}\right)=\mu_{1}R, & C{|}_{\partial \text{Ω}=0}=0, \end{array}$$

• Where _Ф_ is the solution of


$$-K{\text{Δ}}_{x}\text{Φ}=\sigma -\frac{1}{\left|\Omega \right|}\int_{\text{Ω}}\sigma \left(y\right)\text{ d}y,$$

• Endowed with the homogeneous Neumann boundary condition, together with the constraint


(12) $$\int_{\text{Ω}}\delta \left(x\right)C\left(x\right)\text{ d}x=\lambda .$$

This can be interpreted as an implicit definition of the total mass *μ*_1_ to be the value such that the solution of the boundary value problem (7) satisfies (8): in other words, it defines implicitly the mass of the residual tumor *μ*_1_ to be the value such that the solution of the stationary boundary value problem for *C* defines a death rate that exactly compensates the exponential growth rate of the growth division equation. The existence of an equilibrium state defined in this way is rigorously justified in (10, Theorem 2).

Theorem 2.1. Let *x*↦*R*( *x* )∈*L* ^2^ ( Ω ) be a non negative function. If *λ*>0 is small enough, there exists a unique *μ*_1_ ( *λ* )>0 such that the solution *C*_*μ*_1_ ( *λ* )_ of the stationary equation (7) satisfies (8).

Theorem 2.1 requires a smallness assumption; for (2) with constant growth rate *V* and division rate *a*, this is a smallness assumption on *a*. Numerical experiments have shown different large time behaviors for the initial-boundary value problem (1a)-(1e) (an example will be presented later on):

- When the source term *R* is space-homogeneous, the expected behavior seems to be very robust. The immune cell concentration tends to fulfill the constraint ${\bar{\mu }}_{c}\left(t\right)∼\lambda$ as time becomes large, and the size repartition of tumor cells tends to the eigenfunction $\bar{N}$ . The total mass *μ*_1_ tends to a constant; however the asymptotic value cannot be predicted easily.
- When *R* has space variations, the asymptotic behavior seems to be much more sensitive to the parameters of the model, in particular to the aggressiveness of the tumor (characterized by the cell division rate *a*). On short time scale of simulations, we observe alternance of growth and remission phases, and the damping to the equilibrium could be very slow.

These observations bring out the complementary roles of different type of cytotoxic cells (45). The NK cells could be seen as a space-homogenous source of immune cells, immediately available to fight against the tumor, at the early stage of tumor growth. In contrast, T-cells need an efficient priming which occurs in the draining lymph nodes, and their sources is therefore non-homogeneously distributed. Eventually, NK and CD8^+^ T-cells cooperate to the anti-tumor immune response.

**Figure 3:**
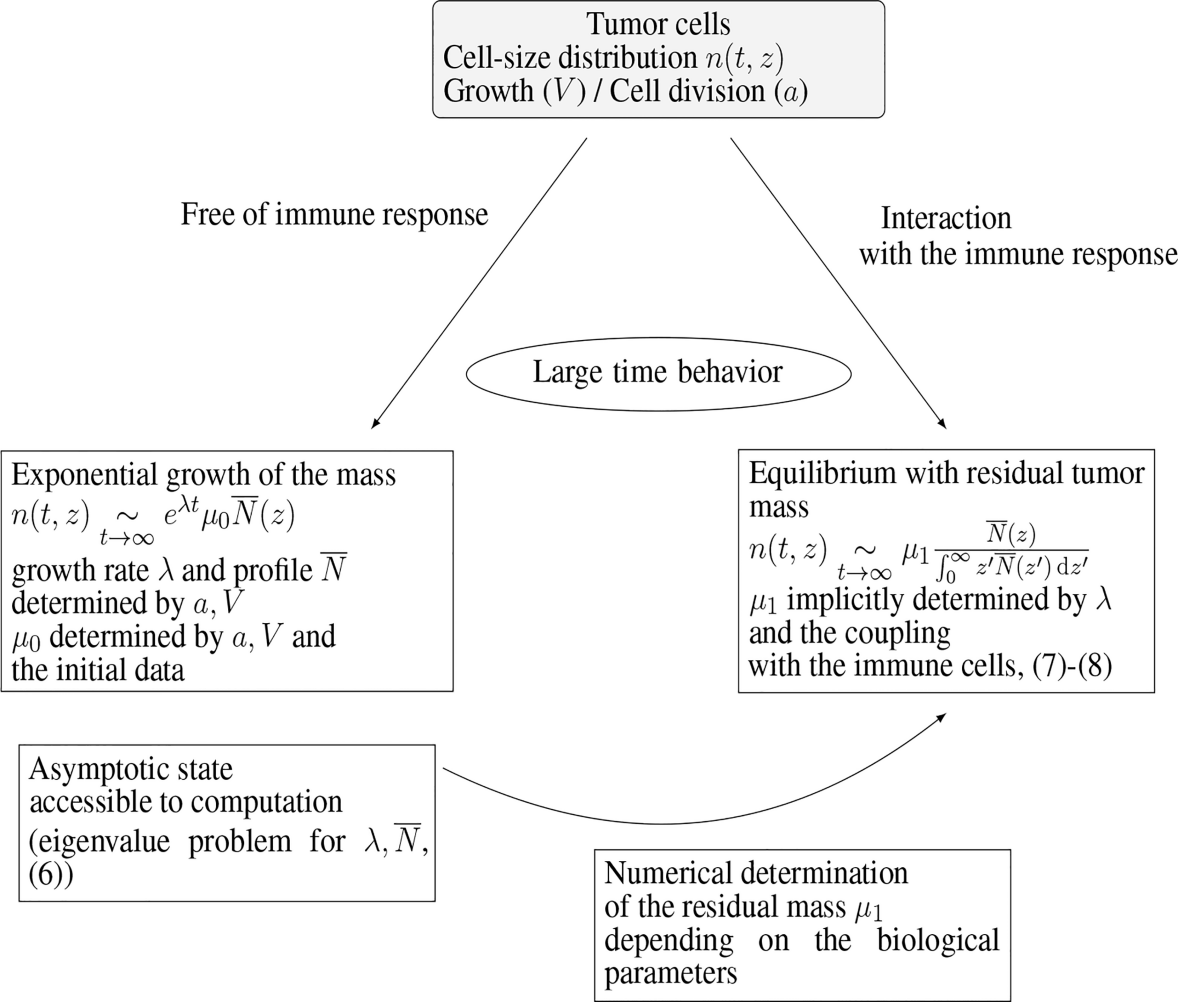
Connection of the equilibrium state with the eigenstate of the growth-division equation, and interpretation of the residual tumor mass.

Numerical experiments thus show that the model (1a)–(1e) is able to reproduce, in the long-time range, cancer-persistent equilibrium, but the features of the equilibrium, and its ability to establish, are highly sensitive to the parameters. To discuss this issue further, we focus here on the mass at equilibrium considered as a critical quantity that evaluates the efficacy of the immune response. Indeed, it is known that a tumor gains in malignancy when its mass reaches certain thresholds (45, 46). The smaller the tumor mass at equilibrium, the better the vital prognosis of the patient. In doing so, we do not consider transient states and time necessary for the equilibrium to establish. The interest of the interpretation of the equilibrium by means of an eigenproblem relies on the fact that the equilibrium state can be determined a priori, at least through numerical simulations, without running the initial boundary value problem over long time ranges: given a set of biological parameters it can be obtained by solving the eigenvalue problem for $\left(\lambda ,\bar{N}\right)$ and the constrained stationary drift-diffusion equation for *C*, see **Figure 3**. In turn, since the equilibrium state can be computed at low numerical cost, a wide range of parameters can be considered and the role of the parameters can be investigated in details. The determination, on numerical grounds, of the equilibrium state relies on a two-step process, as schematised in **Figure 3**. First, we compute the normalized eigenstate of the tumor cell equation, second, we find the tumor mass which makes the coupled death rate fit with the eigenvalue. To this end, we have developed a specific numerical approach.

#### 2.1.2 The Eigen-Elements of the Growth-Division Equation

The numerical procedure is fully detailed and analyzed in Appendix A; it is inspired from the spectral analysis of the equation: λ is found as the leading eigenvalue of a conveniently shifted version of the growth-division operator. In practice, we work with a problem where the size variable is both truncated and discretized. Hence, the problem recasts as finding the leading eigenvalue of a shifted version of the underlying matrix, which can be addressed by using the inverse power method [(47), Section 1.2.5]. We refer the reader to (48, 49) for a thorough analysis of the approximation of eigenproblems for differential and integral operators, which provides a rigorous basis to this approach. It is also important to check a priori, based on the analysis of the equation (32), how large the shift should be, and that it remains independent on the numerical parameters. As already mentioned, for some specific division and growth rates, the eigenpair $\left(\lambda ,\bar{N}\right)$ is explicitly known, see (32). We used these formula to validate the ability of the algorithm to find the expected values and profiles.

#### 2.1.3 Computation of the Equilibrium Mass

Having at hand the eigenvalue λ, we go back to the convection-diffusion equation (7) and the constraint (8) that determine implicitly the total mass *μ*_1_ of the residual tumor. For a given value of *μ*_1_, we numerically solve (7) by using a finite volume scheme, see (10, Appendix C). Then, we use the dichotomy algorithm to fit the constraint:

- The chemo-attractive potential _Ф_ is computed once for all.
- Pick two reference values 0 < μ*_a_* < μ*_b_*; the mass we are searching for is expected to belong to the interval (μ*_a_*, μ*_b_*).
- Set $\mu_{1}=\frac{\mu_{a}+\mu_{b}}{2}$ and compute the associated solution *C*_*μ*_ 1__ of (7) (the subscript emphasizes the dependence with respect to *μ*_1_). Evaluate the discrete version of the quantity *I*=∫^​^ *δC*_*μ*_1__  d*x*−*λ*
- If *I* < 0, then replace *μ_a_* by *μ*_1_, otherwise replace *μ_b_* by *μ*_1_.
- We stop the algorithm when the relative error $\frac{\mu_{b}-\mu_{a}}{\mu_{a}}<\varepsilon$ is small enough.

It is also possible to design an algorithm based on the Newton method. However, this approach is much more numerically demanding (it requires to solve more convection-diffusion equations) and does not provide better results.

### 2.2 Identification of Biological Parameters

In order to go beyond the qualitative discussion of (10), the model should be challenged with biological data. The PDE system (1a)-(1e) is governed by the set of parameters collected in **Table 1**. Most parameter values were retrieved from previously published experimental results and we propose an estimation of the remaining parameters *R, a, V* based on the experimental study performed in (59) where the development of chemically-induced cutaneous squamous cell carcinoma (cSCC) is investigated.

**Table 1:** Key model parameters and their biophysical meaning.

Symbol	Description	Value and unit	References
*X*	chemotactic coefficient	8.64×10^1^−8.64×10^6^ *mm* ^2^·*mmol* ^−1^·*day* ^−1^	(Macrophages) (50)
*D*	natural space diffusion coef. of the cytotoxic effector cells population	8.64×10^−5^−10^−3^ *mm* ^2^·*day* ^−1^	(CD8^+^ T-cells) (23, 51)
*R*	the normal rate of influx of effector immune cells	$\text{log}\left(R\right)∼N\left(\text{log}\left(2.2\times 10^{-6}\right),0.84\right)\left(\frac{cell_{n}\cdot mm^{-3}}{cell_{n}\cdot \mu m^{3}}\cdot day^{-1}\right)$	estimated
*γ*	natural death rate of the tumor antigen-specific cytotoxic effector cells	2×10^−2^−1 *day* ^−1^	(14, 20, 22, 52)
*A*	strength of the immune response	$2-57.6\;cell_{n}^{-1}\cdot day^{-1}$	(53–56)
*K*	diffusion coefficient for the attractive potential *ϕ*	10^−2^−1 *mm* ^2^·*day* ^−1^	(23, 57)
*A_σ_*	strength of the chemical signal induced by each tumor cell	5·10^−17^−0.625×10^−16^ *mmol*·^−1^ *μm* ^3^·*day* ^−1^	(58)
*a*	division rate of the tumor cells	log(*a*)∼*N*(log(0.12),0.2)(*a* in *day* ^−1^)	estimated
*V*	growth rate of the tumor cells	log(*V*)∼*N*(log(816.33),0.51)(*V* in *μm* ^3^·*day* ^−1^)	estimated

Calibrating the parameters of the equations is an issue due to the lack of direct measurements, and the fact that experimental data are obtained at the price of the sacrifice of mice. Consequently, beyond the cost of the experiments, it also means that a time evolution of the quantities of interest is usually not affordable. Therefore, a specific procedure should be developed in order to estimate the parameters from the experimental data points. Since the informations on the parameters are quite poor, we restrict to the case where the coefficients *a, V, R* are constant, which is also a reasonable assumption when dealing with the earliest stages of the tumor development. In order to identify the parameters, we shall use a degraded version of the equations.

Neglecting the immune response, the tumor growth is driven by (5). As explained above, this leads to an exponential growth of the tumor mass, see (32–34, 44). Let $t\mapsto \mu_{0}\left(t\right)=\int_{0}^{\infty }n\left(t,z\right)\text{ d}z$ , the total number of tumor cells, and $t\mapsto \mu_{1}\left(t\right)=\int_{0}^{\infty }zn\left(t,z\right)\text{ d}z.$

Integrating (9) with respect to size variable, with integration by parts, and bearing in mind that the cell division operator is mass preserving, we thus get


(9) $$\begin{array}{l} \;\\ \begin{array}{cc} \frac{d}{dt}\mu_{0}=a\mu_{0}, & \frac{d}{dt}\mu_{1}=V\mu_{0}. \end{array} \end{array}$$

Next, assuming space homogeneity of the immune cells concentration and neglecting the displacement and the natural death rate of the immune cells, the immune cells concentration is driven by


(10) $$\frac{d}{dt}c=R\mu_{1}$$

Based on this simplified dynamics, reduced to (9)-(10), we used the Nonlinear Mixed Effects Modeling (NMEM) in order to estimate the parameters *a, V, R* from the experimental data. Let *N* denote the number of mice within the population and $Y_{i}^{\left(k\right)}=\left\{y_{i1}^{\left(k\right)},\cdots ,y_{in^{i}}^{\left(k\right)}\right\}$ the vector of longitudinal measurements for the *i*th mouse: $y_{ij}^{\left(k\right)}$ is a typical observation of the mouse *i* for a given measurement type *k* ∈ { 0,1,2} (with (0, 1, 2) referring to ( *μ*_0_ ,*μ*_1_ ,*c* ) respectively) at time $t_{ij}^{k}$ for *i* ∈ { 1, … ,*N* } and *i* ∈ { 1, … ,*N* } . We suppose that the statistics of the measurements obeys, for $k\;\in \;\left\{0,\;1,\;2\right\},\;j\;\in \;\left\{1,\;\ldots \;n_{i}^{k}\right\},\;i\;\in \;\left\{1,\;\ldots \;,N\right\}$


(11) $$y_{ij}^{\left(k\right)}=f^{\left(k\right)}\left(t_{ij}^{k};\theta_{i}^{k}\right)+e_{ij}^{\left(k\right)},$$

where $f^{\left(k\right)}\left(t_{ij}^{k};\theta_{i}^{k}\right)$ is the evaluation of the model at time $t_{ij}^{k},\theta_{i}^{k}\in {\mathbb{R}}^{p}$ is the vector of the parameters describing the individual *i* and $e_{ij}^{\left(k\right)}$ the residual error model. The inter-individual variability is described by the combination of fixed effects , which, by definition, are constant within the population and along time, and random effects $\eta_{i}^{k}$ which explain the inter-individual variability among the mice. The positivity of the parameters is ensured by assuming that the individual parameters follow a log-normal distribution. In other words, the random effects are normally distributed with mean zero and a variance-covariance matrix *W*. For instance *W*=diag( *ω* ^0^ ,*ω* ^1^ ,*ω* ^2^ ) where the *ω* ^*k*^’s stand for the variance of the parameters *a, V, R*. Therefore, we have


(12) $$\begin{array}{cc} \log\;\theta_{i}^{k}=\log\;(\theta_{pop}^{k})+\eta_{i}^{k}, & \eta_{i}^{k}∼N\left(0,\omega^{k}\right) \end{array}$$

for *k*∈{ 0,1,2 } . The error model is assumed to be proportional to the model evaluation and is defined as follows:


(13) $$e_{ij}^{\left(k\right)}=\left(b^{\left(k\right)}f^{\left(k\right)}\left(t_{ij}^{k};\theta_{i}^{k}\right)\right)\epsilon_{ij}$$

Where *ϵ*_*ij*_ ∼*N*( 0,1 ) represents the statistical model residual errors and *b* ^(^ *^k^* ^)^ is the proportionality factor measuring the relative amplitude of the errors.

#### 2.2.1 Estimation of the Model Parameters

According to the experimental procedure in (59), 5× 10^5^ mSCC38 were injected to each mouse at time t_0_ = 0. Therefore we fixed the initial number of tumor cells to *μ*_0_ ( 0 )=5× 10^5^ *cells*. Assuming that each tumor cell is spherically shaped with a radius 15 μ m, we set *μ*_1_ ( 0 )=7.1*mm* ^3^. The initial concentration of immune cells is fixed to *c*_0_ = 0: we suppose that initially there is no effector immune cells (or at least it means that the initial concentration of activated immune cells is negligible compared to the concentration of resting cells). Some data points were censored due to the sacrifice of the individual for flow cytometry cell counting. The censored data points have been handled by Limit Of Quantification (LOQ) censoring (60). Let $I_{ij}^{k}$ be the finite or infinite censoring interval for mouse *i*, measurement *k* and time $t_{ij}^{k}$ and


$$\mathbb{P}(y_{ij}^{\left(k\right)}\in I_{ij}^{k}|\theta_{i}^{k})=\int_{I_{ij}^{k}}p_{y_{ij}^{\left(k\right)}|\theta_{i}^{k}}(x|\theta_{i}^{k})\text{ d}x,$$

where $p_{y_{ij}^{\left(k\right)}|\theta_{i}^{k}}$ is the conditional distribution of $y_{ij}^{\left(k\right)}$ given $\theta_{i}^{k}$ . Let us collect in a vector *α*=( *a*_*pop*_,*V*_*pop*_, *R*_*pop*_, *ω*_*a*_, *ω*_*V*_, *ω*_*R*_, *b*_*a*_, *b*_*V*_, *b*_*R*_) the parameters of the model; they are estimated by maximizing the observed likelihood function


(14) $$\begin{array}{l} ℒ\left(\alpha ,y\right)=\prod_{k=0}^{2}\prod_{i=1}^{N}\prod_{j=1}^{n_{i}^{k}}\int p{\left(y_{ij}^{\left(k\right)}|\theta_{i}^{k}\right)}^{1_{\left\{y_{ij}^{\left(k\right)}\notin I_{ij}^{k}\right\}}}\\ \times \mathbb{P}{\left(y_{ij}^{\left(k\right)}\in I_{ij}^{k}|\theta_{i}^{k}\right)}^{1_{\left\{y_{ij}^{\left(k\right)}\in I_{ij}^{k}\right\}}}p\left(\theta_{i}^{k};\alpha \right)\text{ d}\theta_{i}^{k}. \end{array}$$

To this end, we used the Stochastic Approximation of the Expectation Maximization algorithm (SAEM) implemented in the MONOLIX R API (61). Furthermore, the individual parameter estimators $\hat{\theta_{i}^{k}}$ are computed in MONOLIX (61) by means of the Empirical Bayes Estimate (EBE) of $\theta_{i}^{k}$ which corresponds to the mode of the conditional distribution $p\left(\theta_{i}^{k}|y_{i}^{k};\hat{\alpha }\right)$ (where $\hat{\alpha }$ corresponds to estimated parameters).

**Figure 4:**
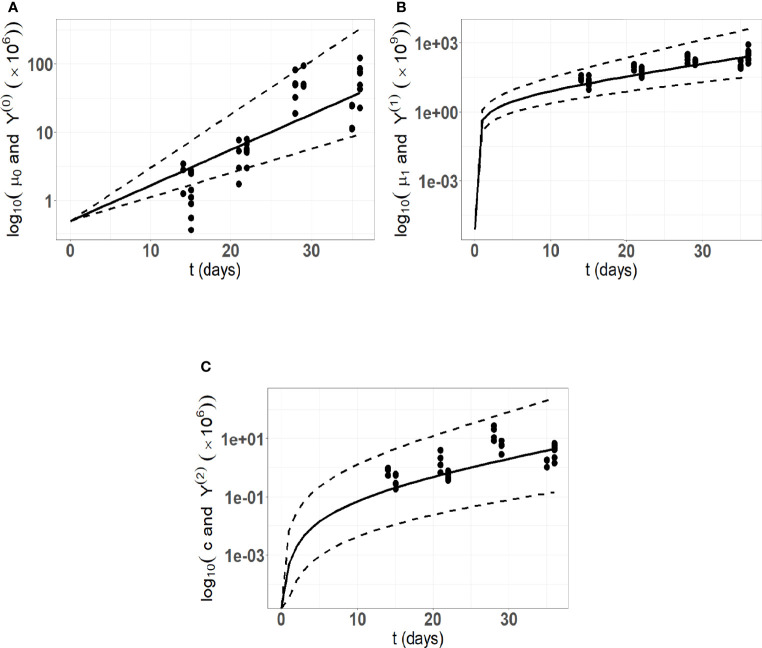
Model fitting to the *in vivo* experimental cSCC tumor growth data. Here, we are using 34 data points from an *in vivo* experimental cutaneous squamous cell carcinoma (cSCC) tumor growth mouse model (59). **(A)** Number of tumor cells kinetics; **(B)** Tumor volume kinetics (*μm* ^3^); **(C)** Concentration of immune cells kinetics. The solid lines represent the model prediction using the mean estimated parameters, the dashed lines represent the model predictions using the 5th and 95th percentiles of the parameters distribution.

A preliminary estimation procedure indicates a significant correlation between the parameters *a* and *R* ( t-test p-value 2.6× 10^−6^) . Hence, introducing this correlation into the variance covariance matrix of the random effects by setting covar ( *a*,*R* )=*ρ*_*aR*_ *ω*_*a*_ *ω*_*R*_ , where *ρ*_*aR*_ represents the correlation coefficient between *a* and *R*, enhances the goodness of fit. The estimated value of *ρ*_*aR*_ is 0.8 with a relative standard error of 13%. The parameters in *a* were estimated with reasonable standard errors (computed using the stochastic approximation) and relative standard errors (max (*R.S.E*) = 30.6 and min (*R.S.E.*) = 3) which indicate that the model parameters are identifiable. The ShapiroWilk test reinforces the normality hypotheses on the random effects $\eta_{i}^{\left(k\right)}$ (the p-values for *η*_*a*_ ,*η*_*V*_  and *η*_*R*_ are respectively 0.83, 0.61, 0.2). Pictures indicating the fits are provided in **Figure 4**, and detailed parameter estimates are given in **Table 2**.

**Table 2:** Estimated value of the parameters with their Standard Error (S.E.) and Relative Standard Error (R.S.E).

parameters	value	S.E	R.S.E (%)
*a_pop_*	0.12	0.0041	3
*V_pop_*	816.33	92.59	11
*R_pop_*	2.2×10^−6^	3.6×10^−7^	16
*ω_a_*	0.20	0.027	13.5
*ω_V_*	0.51	0.075	15
*ω_R_*	0.84	0.11	13
*b_a_*	0.37	0.041	11
*b_V_*	0.17	0.052	31
*b_R_*	0.18	0.056	30
*ρ_aR_*	0.8	0.1	13

### 2.3 Materials

#### 2.3.1 Mice

FVB/N wild-type (WT) mice (Charles River Laboratories, St Germain Nuelles, France) were bred and housed in specific-pathogen-free conditions. Experiments were performed using 6-7 week-old female FVB/N, in compliance with institutional guidelines and have been approved by the regional committee for animal experimentation (reference MESR 2016112515599520; CIEPAL, Nice Côte d’Azur, France).

#### 2.3.2 *In Vivo* Tumor Growth

mSCC38 tumor cell line was established from DMBA/PMA induced sSCCs and maintained in DMEM (Gibco-ThermoFisher Scientific, Courtaboeuf, France) supplemented with 10% heat-inactivated fetal bovine serum (FBS) (GE Healthcare, Chicago, Illinois, USA) penicillin (100 *U/ml*) and streptomycin (100 *μg/ml*) (Gibco-ThermoFisher Scientific, Courtaboeuf, France). 5× 10^5^ mSCC38 were intradermally injected in anesthetized mice after dorsal skin shaving. Tumor volume was measured manually using a ruler and calculated according to the ellipsoid formula: Volume=Length(*mm* )×Width( *mm* )×Height( *mm* )×*π*/6.

#### 2.3.3 Tissue Preparation and Cell Count

mSCC38 were excised and enzymatically treated twice with collagenase IV (1*mg*/*ml*) (Sigma-Aldrich, St Quentin Fallavier, France), and DNase I (0.2 *mg*/*ml*) (Roche Diagnostic, Meylan, France) for 20 minutes at 37°*C* . Total cell count was obtained on a Casy cell counter (Ovni Life Science, Bremen, Germany). Immune cell count was determined from flow cytometry analysis. Briefly, cell suspensions were incubated with anti-CD16/32 (2.4G2) to block Fc receptors and stained with anti-CD45 (30-F11)-BV510 antibody and the 7-Aminoactinomycin D (7-AAD) to identify live immune cells (BD Biosciences, Le Pont de Claix, France). Samples were acquired on a BD LSR Fortessa and analyzed with DIVA V8 and FlowJo V10 software (BD Biosciences, Le Pont de Claix, France).

#### 2.3.4 Mathematical and Statistical Analysis

Computations were realized in Python and we made use of dedicated libraries, in particular the gmsh library for the computational domain mesh generation, the packages optimize (for the optimization methods using the Levenberg-Marquard mean square algorithm; similar results have been obtained with the CMA-ES algorithm of the library cma) from the library scipy, the MONOLIX R API and application for the model calibration to the experimental data (61), the library Pygpc for the generalized Polynomial Chaos approximation (62) and the library Salib for the sensitivity analysis (63).

## 3 Results

### 3.1 Validation of the Method

For all the simulations discussed here, we adopt the same framework as in (10): the tumor is located at the origin of the computational domain Ω, which is the two-dimensional unit disk. Otherwise explicitly stated, we work with the lower bound of the parameters collected in **Table 1**. When necessary, the initial values of the unknowns are respectively *μ*_0_ (0) = 1 *cell_n_*, ue *μ*_1_ (0) = 14137.2 *μm* ^3^, *c*(0,*x*) = 0.

**Figure 5:**
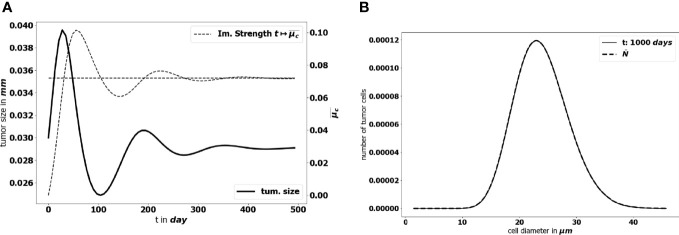
**(A)** Time evolution of the diameter of the tumor (bold black line) and concentration of active immune cells (dotted gray line). The predicted asymptotic value for the latter is represented by the horizontal dotted line. **(B)** Comparison of the tumor cell-size distribution at *t* = 1000 *days* with the positive eigenstate of the cell division equation (x-axis: size of the tumor cells, y-axis: number of tumor cells at the final time). For this simulation Ω={ ∥ *x* ∥≤1 } , the data are given by the lower bound of the parameters collected in **Table 1** and ( *a*,*V*,*R* )=( 0.072, 713.61, 1.74× 10^−7^ ).

To start with, we perform a simulation of the initial-boundary value problem (1a)-(1e). **Figure 5** illustrates how the equilibrium establishes in time: as time becomes large, the effective concentration of active immune cells, that is denoted


$$\bar{\mu_{c}}\left(t\right)=\int_{\text{Ω}}\delta \left(x\right)c\left(t,x\right)\text{ d}x$$

tends to the eigenvalue of the cell-division equation, the total mass *μ*_1_(*t*) tends to a constant and the size distribution of tumor cells takes the profile of the corresponding eigenstate. This result has been obtained by setting ( *a*,*V*,*R* )=( 0.072, 713.61, 1.74× 10^−7^ ) . We observe a non symmetric shape of the size distribution of tumor cells, peaked about a diameter of 23 *μm*, which is consistent with observational data reporting the mean size distribution of cancer cells (64, 65).

For the simplest model of growth-division with *a* and *V* constant, we know an expression of the eigenstate $\left(\lambda ,\bar{N}\right)$ ; however, we do not know an explicit evaluation of the residual mass. Nevertheless, we can compare the results of the inverse power-dichotomy procedure that predicts the residual mass, to the large time simulations as performed in (10). Let $\mu_{1}^{f}$ be the asymptotic value of the total mass given by the large time simulation of the initial-boundary value problem (and checking that the variation of the total mass has become negligible) and let $\mu_{1}^{pd}$ be the mass predicted by the power-dichotomy procedure. We set


$$E_{\mu_{1}}=\frac{\left|\mu_{1}^{f}-\mu_{1}^{pd}\right|}{\mu_{1}^{f}}.$$

The results for several cell division rates *a* are collected in **Table 3**: the numerical procedures finds the same equilibrium mass as the resolution of the evolution problem, which is a further validation of the method.

**Table 3:** Comparison of the large time tumor mass and the predicted tumor mass for several values of *a*.

*a*	$\mu_{1}^{f}$ (*mm* ^3^) at final time *T* = 500	$\mu_{1}^{pd}$ (*mm* ^3^)	*E*_*μ*_ 1__
0.103	7.67271875 × 10^-5^	7.67271872 × 10^-5^	4.10 × 10^-9^
0.15	1.11701535 × 10^-4^	1.11701543 × 10^-4^	7.97 × 10^-8^
0.20	1.48924575 × 10^-4^	1.48924641 × 10^-4^	4.40 × 10^-7^
0.3	2.23420663 × 10^-4^	2.23420562 × 10^-4^	4.53 × 10^-7^
0.351	2.61368442 × 10^-4^	2.61367974 × 10^-4^	1.80 × 10^-6^

Further validation concerning the ability in finding the leading eigenstate are presented in Appendix A. The method has been successfully employed to predict equilibrium state when dealing with complex growth rate and division operator in (42).

### 3.2 Numerical Simulations Show How Parameters Influence Equilibrium

The numerical methods were next used to assess how the parameters influence the equilibrium. In particular, we wish to assess the evolution of the tumor mass at equilibrium according to immune response and tumor growth parameters.

For the numerical simulations presented here, we thus work on the eigenproblem (6) and on the constrained system (7)-(8). Unless precisely stated, the immune response parameters are fixed to the lower bounds in **Table 1**. The tumor growth parameters are set to = 0.1 *day* ^-1^, *V* = 713.61 *μm* ^3^ *day* ^-1^ and $R=1.74\times 10^{-7}\frac{cell_{n}\cdot mm^{-3}}{cell_{n}\cdot \mu m^{3}}\cdot day^{-1}$ .

The main features of the solutions follow the observations made in (10), which were performed with arbitrarily chosen values for the parameters. We observe that ${\bar{\mu }}_{c}\left(t\right)=\int_{\text{Ω}}\delta \left(y\right)c\left(t,y\right)\text{ d}y$ tends to the division rate *a*, which in this case corresponds to the leading eigenvalue of the cell-division equation. It is important to note that the predicted diameter of the tumor at equilibrium — see **Figure 5** — is significantly below modern clinical PET scanners resolution limit, which could detect tumors with a diameter larger than 7*mm* (66). This is consistent with the standard expectations about the equilibrium phase (3), but, of course, it makes difficult further comparison of the prediction with data.

**Figure 6:**
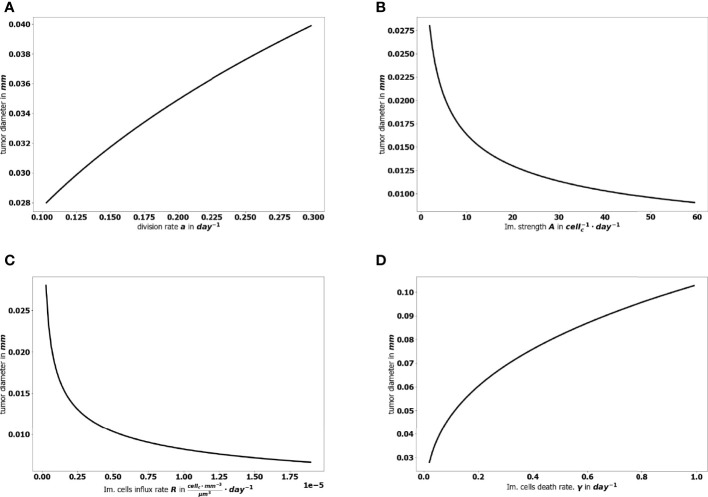
Evolution of the tumor diameter at equilibrium, with respect to **(A)** the division rate of tumor cells *a*, **(B)** the strength of the effector immune cells *A*, **(C)** the influx rate of effector immune cells *R*, **(D)** the natural death rate *γ* of the effector cells.

The aggressiveness of the tumor is characterized by the division rate, the variations of which impact the size of the tumor at equilibrium: the larger *a*, the larger the residual tumor, see **Figure 6A**. Increasing the immune strength *A* increases the efficacy of the immune response, reducing the size of the residual tumor see **Figure 6B**. Similarly, increasing the mean rate of influx of effector immune cells in the tumor microenvironment *R*, decreases the tumor size at equilibrium, see **Figure 6C**. On the contrary, increasing the death rate of the immune cells *γ* reduces the efficacy of the immune response and increases the equilibrium tumor size see **Figure 6D**.

**Figure 7:**
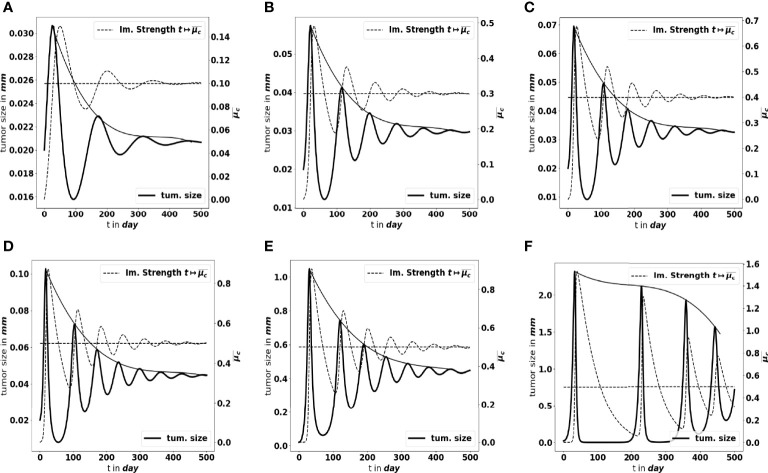
Large-time simulation of the PDE system: evolution of the tumor diameter (bold black line, left axis), and of the concentration of immune cells ${\bar{\mu }}_{c}$ (dotted grey line, right axis), for several values of the division rate *a*: **(A)** *a* = 0.1 *day* ^-1^, **(B)** *a* = 0.3 *day* ^-1^, **(C)** *a* = 0.4 *day* ^-1^ and for several values of the immune strength *A*: **(D)** $A=1\;cell_{c}^{-1}\cdot day^{-1}$ , **(E)** $A=1\cdot 10^{-3}\;cell_{c}^{-1}\cdot day^{-1}$ , **(F)** $A=1\cdot 10^{-5}cell_{c}^{-1}\cdot day^{-1}$ . The horizontal dotted line represents the predicted asymptotic value for ${\bar{\mu }}_{c}$ . The solid line gives the envelope of the oscillations, indicating a polynomial damping rate. The equilibrium needs more time to establish as the strength of the immune system decreases.

Moreover, as mentioned above, not only the parameters determine the equilibrium mass, but they also impact how the equilibrium establishes. **Figures 7A–C** shows what happens by making the tumor cell division rate *a* vary. There are more oscillations along time, with larger amplitude, as *a* increases. Similar observations can be made when reducing the strength of the immune system *A* (likely out of its realistic range), see **Figures 7D–F**. The smaller *A*, the weaker the damping of the oscillations and the longer the periods. We notice that the decay of the maximal tumor radius holds at a polynomial rate. In extreme situations, either the damping is very strong and the equilibrium establishes oscillation-free or the equilibrium does not establish on reasonable observation times, and the evolution can be confounded with a periodic alternance of growing and remission phases. Such scenario illustrates that the relevance of the equilibrium can be questionable depending on the value of the parameters. In what follows, we focus on the details of the equilibrium itself, rather than on the transient states.

### 3.3 Global Sensitivity Analysis on the Equilibrium Mass Identifies the Key Parameters to Target in Cancer Therapy

Since the equilibrium state can be computed for a reduced numerical cost (it takes about 1/4 of a second on a standard laptop), we can perform a large number of simulations, sampling the range of the parameters. This allows us to discuss in further details the influence of the parameters on the residual mass and, by means of a global sensitivity analysis, to make a hierarchy appear according to the influence of the parameters on this criterion. Ultimately, this study can help in proposing treatments that target the most influential parameters.

Details on the applied methods for the sensitivity analysis can be found in Appendix B. Among the parameters, we distinguish:

- The tumor cell division rate *a* which drives the tumor aggressiveness,
- The efficacy of the immune system, governed by the mean influx rate of activated effector immune cells *R*, the strength of the immune response *A*, the chemotactic sensitivity χ, the death rate *γ* of the immune cells, and the strength of the chemical signal induced by each tumor cell *A_σ_*
- Environmental parameters such as the diffusion coefficients *D* (for the immune cells) and *K* (for the chemokine concentration).

We assume that the input parameters except *a* and *R* are independent random variables. Due to the lack of knowledge on the specific distribution of most of the parameters, the most suitable probability distribution is the one which maximizes the continuous entropy (67), more precisely, the uniform distribution over the ranges defined in **Table 1**. Therefore, the uncertainty in the parameter values is represented by uniform distributions for the parameters ( *A*,*χ*,*D*,*A*_*σ*_ ,*γ*,*K* ) and by log-normal distributions for the parameters *a* and *R*. In what follows, the total mass at equilibrium, *μ*_1_, given by the power-dichotomy algorithm, is seen as a function of the uncertain parameters:


(15) $$\mu_{1}=f\left(a,A,R,\chi ,D,A_{\sigma },\gamma ,K\right)$$

**Figure 8:**
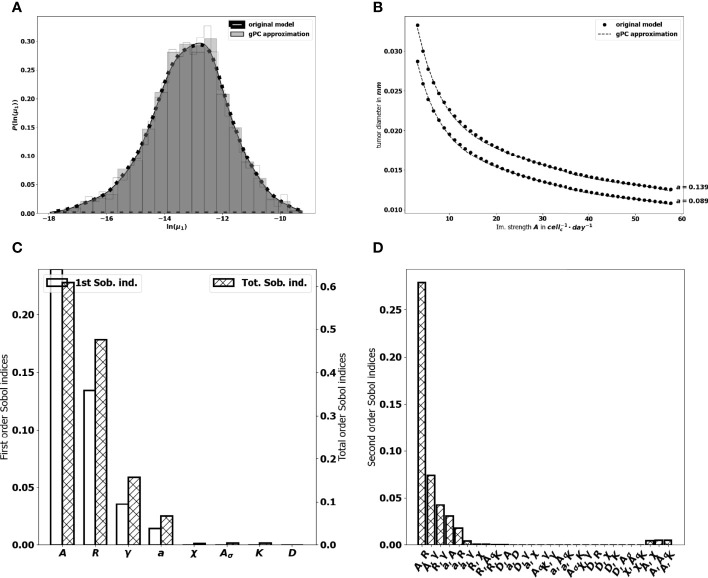
**(A)** comparison between the pdf of In (*μ*_1_) from the gPC approximation and the pdf from the original model. **(B)** Comparison between the value of *μ*_1_ generated by the power-dichotomy algorithm and the gPC approximation. **(C)** First (empty, left scale) and total (dashed, right scale) order Sobol indices for *μ*_1_. **(D)** Second order Sobol indices for *μ*_1_.

To measure how the total variance of the output *μ*_1_ of the algorithm is influenced by some subsets *i*_1_ ⋯*i*_*p*_ of the input parameters *i*_1_ ⋯*i*_*p*_ ( *k*≥*p* being the number of uncertain input parameters), we compute the so-called Sobol’s sensitivity indices. The total effect of a specific input parameter *i* is evaluated by the total sensitivity index $S_{T}^{\left(i\right)}$ , the sum of the sensitivity indices which contain the parameter *i*. (Details on the computed Sobol indices can be found in Appendix B). The computation of these indices is usually based on a Monte Carlo (MC) method [see (68, 69)] which requires a large number of evaluations of the model due to its slow convergence rate $\left(O(1/\sqrt{N}\right)$ where *N* is the size of the experimental sample). To reduce the number of model evaluations, we use instead the so-called generalized Polynomial Chaos (gPC) method [see (70)]. The backbone of the method is based on building a surrogate of the original model by decomposing the quantity of interest on a basis of orthonormal polynomials depending on the distribution of the uncertain input parameters *θ*( *ω* )=( *a*,*A*,*R*,*χ*,*D*,*A*_*σ*_ ,*γ*,*K* ), where *ω* represents an element of the set of possible outcomes. Further details on the method can be found in (71). For uniform distributions, the most suitable orthonomal polynomial basis is the Legendre polynomials. The analysis of the distribution of *μ*_1_ after a suitable sampling of the parameters space indicates that *μ*_1_ follows a log-normal distribution. This distribution is not uniquely determined by its moments (the Hamburger moment problem) and consequently cannot be expanded in a gPC [see (72)]. Based on this observation, to obtain a better convergence in the mean square sense, we apply the gPC algorithm on the natural logarithm of the output *μ*_1_. Typically, ln(*μ*_1_) is decomposed as follows:


(16) $$\text{ln }\left(\mu_{1}\left(\omega \right)\right)=\sum_{\alpha \in J_{k,p}}q_{\alpha }L_{\alpha }\left(\theta \left(\omega \right)\right)+\epsilon ,$$

where *ϵ* corresponds to the approximation error, $J_{k,p}=\left\{\alpha \in {\mathbb{N}}^{k}:\sum_{i=1}^{k}\alpha_{i}\leq p\right\}$ and *p* represents the highest degree of the expansion. Hence, the dimension of the polynomial basis is given by $\frac{\left(k+p\right)!}{k!p!}$ . We reduce the number of model evaluations to 642 runs by constraining also the parameters interaction order to 2. For our purpose, a degree *p* = 5 gives a better fit (see **Figures 8A, B** to the original model and the goodness of fit of the gPC algorithm is measured by a Leave One Out Cross Validation (LOOCV) technique (73). The resulting LOO error indicates 0.4% prediction error. The Sobol’s sensitivity indices are then computed from the exponential of the surrogate model (16) by using Monte Carlo simulations combined with a careful space-filling sampling of the parameters space [see (68, 74)]. For the computations, a sample with *N*=1.8× 10^6^ points has been used in order to get stable second order Sobol indices. Indeed, the sensitivity indices that are needed to discriminate the impact of the input parameters are the first and total Sobol’ sensitivity indices. Here, the analysis revealed a significant difference between some first order Sobol’ indices and their corresponding total Sobol indices, which indicated the importance of computing also the second order Sobol’ indices.

It is important to stress that the obtained results, and the associated conclusions, could be highly dependent on the range of the parameter values. This observation makes the measurement/estimation of the parameters a crucial issue which can be dependent on the type of cancer analyzed.

#### 3.3.1 Efficacy of the Immune Response

The first order Sobol indices represented in **Figure 8C** indicate that the parameters which impact the most the variability of the immune-controlled tumor mass at equilibrium are:

- The strength of the lethal action of the immune cells on the tumor cells *A*, by far the most influential, and three additional parameters
- The influx rate of activated effector immune cells into the tumor microenvironment *R*.
- The natural death rate *γ* of the effector immune cells,
- And the division rate *a* of the tumor cells.

This result is consistent with the observations made from the numerical experiments above and in (10), showing a prominent role of the immune response which can be enhanced by increasing either *A* or *R*, and decreasing *γ*. That *A* is the most influential parameter is not that surprising but it is remarkable how far its importance exceeds that of the other parameters. It is also puzzling to see that the chemotactic sensitivity *χ*, like the strength of the chemical signal induced by each tumor cell *A_σ_*, the space diffusion coefficient of the effector immune cells *D* and the diffusion coefficient of the chemokines *K* , have a negligible influence on the immune-controlled tumor mass, see **Figure 8C**, whether individually or in combination with other parameters. This result is consistent with the necessity for immune cells to be able to effectively kill the tumor cells once they reach the tumor site. The second order Sobol’ indices indicate that the leading interactions are the pairs (*A, R*), (*A,γ*), (*R, γ*), (*a,A*), (*a, R*) and (*a, γ*). Accordingly, in order to enhance the immune response, an efficient strategy can be to act simultaneously on the immune strength *A* together with the influx rate of activated immune effector cells *R*. Increasing such influx into the tumor microenvironment by enhancing the activation/recruitment processes leading to the conversion of naive immune cells into activated immune cells potentiate anti-tumor immune responses. Besides, the natural death rate *γ* of the effector immune cells combined to *A* and *R* have an impact, as well as *A* combined with the division rate of the tumor cells, *a*.

#### 3.3.2 The Tumor Aggressiveness

The tumor aggressiveness is mainly described by the cell division rate *a*. The first order Sobol indice indicates that *a* influences significantly the tumor mass at equilibrium, and we observe that the total Sobol index of *a* is higher than the individual one. This indicates that this parameter has strong interactions with the others. By taking a look at **Figure 8D** we remark that *a* interacts significantly with the parameters *A, R, γ*. However, the most significant interaction is the one with *A*. This suggests that combining therapies targeting tumor and immune cells should be more efficient at maintaining immune-mediated tumor mass dormancy (75).

#### 3.3.3 Towards Optimized Treatments

**Figure 9:**
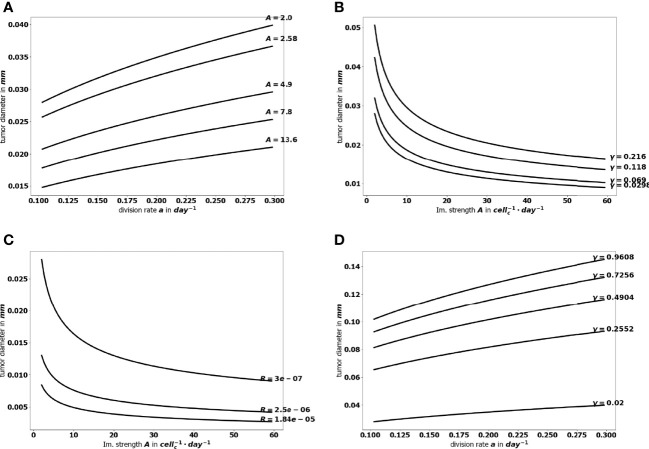
Evolution of the tumor diameter at equilibrium, **(A)** with respect to the division rate *a* for several values of the immune strength *A*, **(B)** with respect to the immune strength *A* for several values of the death rate *γ*, **(C)** with respect to the immune strength *A* for several values of the influx rate of effector immune cells *R*+, and **(D)** with respect to the division rate *a* for several values of the death rate *γ*.

Because equilibrium state can be computed for a reduced numerical cost, it allows a large number of simulation to be performed in a minimal time, so that an extensive sampling of the range of the parameters can be tested. The flexibility of the numerical simulations provides valuable tools to assess the efficiency of a variety of therapeutic strategies and select those that sustain a viable equilibrium and prevent cancer relapses after a surgery or a treatment. **Figure 9** illustrates how the equilibrium mass is impacted when combining variations of two parameters, namely the immune strength *A* combined to the tumor cell division rate *a*, the mean rate of influx of effector immune cells *R* or the death rate of effector immune cells *γ*; and the tumor cell division rate *a* with the death rate *γ*. Interestingly, a reduction of the tumor mass at equilibrium can be obtained significantly more easily by acting on two parameters than on a single one. For instance, reducing the tumor cell division rate *a* from 0.35 to 0.1 cannot reduce the diameter of the tumor below.025 mm, with *A* = 1; while the final size is always smaller when *A* = 3.95. This observation highlights the interest of combined treatments having such complementary actions. The interest is two-fold: either smaller residual tumors can be obtained by pairing two actions, or the same final tumor size can be obtained with a combined treatment having less toxicity than a mono-therapy.

## 4 Discussion

Controlling parameters that maintain immune-mediated tumor mass dormancy is key to the successful development of future cancer therapies. To understand how equilibrium establishes and how it is influenced by immune, environmental and tumor-related parameters, we evaluate the tumor mass which tends to a constant at equilibrium. In this study, we make use of the space and size structured mathematical model developed in (10) to provide innovative, efficient methods to predict, at low numerical cost, the residual tumor mass at equilibrium. By means of numerical simulations and global sensitivity analysis, we identify the elimination rate *A* of tumor cells by immune cells as the most influential factor. Therefore, the most efficient therapeutic strategy is to act primarily on the immune system rather than on the tumor itself. We also demonstrate the need to develop combined cancer treatments, boosting the immune capacity to kill tumor cells (increase *A*), the conversion into efficient immune cells (increase *R*), reducing natural death rate of effector immune cells (decrease *γ*) and reducing the ability of tumor cells to divide (decrease *a*). The combination of such approaches definitely outperforms the performances of a single action; it permits to maintain the tumor in a long-lasting equilibrium state, far below measurement capabilities.

Generally, therapeutic strategies are designed to target preformed, macroscopic cancers. Indeed, patients are diagnosed once their tumor is established and measurable, thus at the escape phase of the cancer immunoediting process (1). The goal of successful treatments is to revert to the equilibrium phase and ultimately to tumor elimination. Experimental evidence and clinical observations indicate that such equilibrium exists but it is difficult to study and measure, the residual tumor mass being below detection limits (1, 2, 3). It is regarded as “a immune-mediated tumor mass dormancy” when the rate of cancer cell proliferation matches their rate of elimination by immune cells. In human, cancer recurrence after therapy and long periods of remission or detection of low number of tumor cells in remission phases are suggestive of such equilibrium phase. Mathematical models can also be used to provide evidence of such state. The system of partial differential equations proposed in (10) is precisely intended to describe the earliest stages of immune control of tumor growth. Remarkably, while being in the most favorable condition, only taking into account the cytotoxic effector immune cells and no immunosuppressive mechanisms, the model reproduces the formation of an equilibrium phase with maintenance of residual tumor cells rather than their complete elimination. Besides suggesting that elimination may be difficult to reach, this finding also brings out the role of leading parameters that shape the equilibrium features and opens new perspectives to elaborate cancer therapy strategies that reach this state of equilibrium.

To decipher tumor-immune system dynamics leading to equilibrium state, we have developed here computational tools. The total mass of the tumor is a critical criterion of the equilibrium and was used to predict parameters that contribute the most to the establishment of the equilibrium. By means of global sensitivity analysis, we identified one leading parameter, *A*, and three others, *R*, *γ* and *a* that affect the most the variability of the immune-controlled tumor mass; *A*, *R* and *γ* are related to immune cells, and *a* to tumor cells. Moreover, the influence of the leading parameters is significantly increased when they are paired. This observation supports the development of combined therapeutic treatments which would be more efficient at reducing tumor growth and toxicity. Because the pairs (*A, R*), (*A, γ*), (*R, γ*), (*A, a*), (*a, R*) and (*a, γ*) are the most influential, we predict that a combination of drugs enhancing antitumor immune responses with drugs diminishing tumor aggressiveness will be the most efficient. This is consistent with the clinical benefit obtained when chemotherapies reducing the tumor cell division rate *a* are combined with immunotherapies increasing *A* and *R* (75), The parameter *A* which governs the efficacy of the immune system to eliminate tumor cells, is the most influential. This finding is consistent with the observation that “hot” tumors infiltrated with immune cells have better prognostic than “cold” tumors (76) and that the immune cells with the strongest positive impact on patient’s survival are the cytotoxic CD8^+^ T cells (77). It is also in line with the success of ICP which revert immune tolerance triggered by chronic activation and upregulation of exhaustion markers on effector T and NK cells, thus not only increasing the parameter *A* but also *R* (78). The leading role of the parameter *A* is also demonstrated by experimental studies and clinical trials, such as adoptive transfer of CAR-T and CAR-NK cells engineered to attack cancer cells, immunomodulating antibody therapies or cancer vaccines which boost the antitumor immune response (75, 79–81). Finally, our finding that the parameter *γ* is highly influential is confirmed by the administration of cytokines that stimulate and increase effector T and NK cell survival which are efficient at controlling tumor growth (81). Thus, altogether, these experimental and clinical data validate the numerical method.

Interestingly, besides the dominant role of the parameter *A*, only two additional parameters related to immune cells *R*, *γ* seem to have an influence on the tumor mass at equilibrium. These data predict that to enhance the immune response, it is more efficient to increase the rate of influx and conversion of naive immune cells into effector cells (parameter *R*) or to increase the lifespan of immune effectors (parameter *γ*) than to increase chemotaxis as a whole (parameters *χ*, *A_a_*, *K* ). The lack of influence of chemotaxis emphasizes that the localization of immune cells within tumors is necessary but not sufficient. Indeed, the leading influence of the parameters *A, R, γ* stresses the importance of having functional immune cells infiltrating tumors. Overcoming immune suppression is therefore highly relevant in therapeutic strategies.

Targeting Immune-mediated tumor mass dormancy is gaining more and more attention, having been linked to recurrence and metastasis (9, 82). The persistence of undetectable tumor cells after primary tumor resection at the primary site but also their spreading to metastatic niches are major causes of treatment failure. Thus, developing strategies to maintain an equilibrium between these tumor cells and the immune response is crucial. Interestingly, a recent study demonstrated a role of the NK cell reservoir in blocking the reawakening of dormant tumor cells (83). The mechanisms involve IL-15 that drives NK cell proliferation and IFN- *γ* secreted by NK. Therapies boosting NK cell activity like IL-15 superagonists, or engineered NK cell engagers are therefore promising strategies to sustain NK cell-mediated maintenance of tumor dormancy (83, 84).

It is appropriate to finally comment on the limitations of this work and provide new avenues for future research. Firstly, the analysis focuses on the asymptotic state, taking full advantage of its mathematical interpretation which makes it easily computable. However, the transient states and the rate at which the equilibrium becomes observable are simply disregarded, while they are certainly essential for assessing the biological relevance of the equilibrium state. Further analysis is therefore needed in order to understand how the parameters of the model influence the trend to equilibrium. Secondly, the modeling approach is facing contradictory requests: on the one hand, the lack of knowledge on the parameters motivates working with a reduced set of equations, at the cost of considering an “averaged” behavior (say for instance between different types of immune cells); on the other hand, it might be important to keep under consideration many relevant and competing effects of cellular interactions. These issues can be addressed with a better access to biological data and through the development of dedicated methods of parameter identification. This is of course even more important when describing the effects of treatments. Thirdly, the present analysis is limited to an idealized situation in which many important effects have been overlooked. In particular, the immune response can also promote the tumor growth. Considering such immune actions leads to a much more complex dynamical behavior and the possible establishment of an escape phase, as shown in (42). Finally geometrical aspects and heterogeneity are poorly addressed and restrict the relevance of the description to the earliest stages of the tumor development. More complex models, with a full space structuration, should be elaborated in order to obtain a more accurate description of the tumor microenvironment.

## 5 Conclusion

In conclusion, clinical trials have been undertaken quite often on assumptions from acquired knowledge on tumor development and immune responses to cancer cells, but without tools to help the decision-making. The numerical methods developed here provide valuable hints for the design and the optimization of antitumor therapies. The approach is in agreement with published experimental findings and clinical evidence. By adapting the range of the parameters to the biological values, one can more precisely adapt the therapeutic strategies to specific types of tumors. We thus conclude that mathematical modelling combined with numerical validation provide valuable information that could contribute to better stratify the patients eligible for treatments and consequently save time and lives. In addition, it could also help to decrease the burden of treatment cost providing hints on optimized therapeutic strategies.

## 6 Computation of the Eigen-Elements of the Growth-Division Equation

The binary division operator (2) is a very specific case, and for applications it is relevant to deal with more general expressions. Namely, we have


(17) $$Q\left(n\right)\left(t,z\right)=-a\left(z\right)n\left(t,z\right)+\int_{z}^{\infty }a\left(z^{\prime }\right)k(z|z^{\prime })n\left(t,z^{\prime }\right)\text{ d}z^{\prime }.$$

In (21), *a*( *z* ^′^ ) is the frequency of division of cells having size *z* ^′^ , and *k*( *z*∣*z* ^′^ ) gives the size-distribution that results from the division of a tumor cell with size *z* ^′^. What is crucial for modeling purposes is the requirement


$$\int_{0}^{z}z^{\prime }k(z^{\prime }|z)\text{ d}z^{\prime }=z,$$

which is related to the principle that cell-division does not change the total mass


$$\int_{0}^{\infty }zQ\left(n\right)\text{ d}z=0.$$

We refer the reader to (32) for examples of such cell-division operators and the analysis of the eigenvalue problem (6) under quite general assumptions of the growth rate *V*, the frequency *a* and the kernel *k*. Our numerical method can handle such general coefficients.

It is important to bear in mind the main arguments of the proof of the existence-uniqueness of the eigenpair $\left(\lambda ,\bar{N}\right)$ for the growth-division equation. Namely, for Λ large enough we consider the *shifted* operator


$$T_{\text{Λ}}N=\text{Λ}N+\partial_{z}\left(VN\right)+aN-\int_{z}^{\infty }a\left(z^{\prime }\right)k(z|z^{\prime })N\left(z^{\prime }\right)\text{ d}z^{\prime }.$$

Then, we check that the operator *S*_Λ_ which associates to a function *f* the solution *n* of *T*_Λ_ *n* = *f* fulfills the requirements of the Krein-Rutman theorem (roughly speaking, positivity and compactness), see (85). Accordingly, the quantity of interest *λ* is related to the leading eigenvalue of *S*_Λ_. In fact, this reasoning should be applied to a somehow truncated and regularized version of the operator, and the conclusion needs further compactness arguments; nevertheless this is the essence of the proof. In terms of numerical method, this suggests to appeal to the inverse power algorithm, applied to a discretized version of the equation. However, we need to define appropriately the shift parameter Λ. As far as the continuous problem is considered, Λ can be estimated by the parameters of the model (32), but it is critical for practical issues to check whether or not this condition is impacted by the discretization procedure. This information will be used to apply the inverse power method to the discretized and shifted version of the problem.

### 6.1 Analysis of the Discrete Problem

The computational domain for the size variable is the interval [0, R] where *R* is chosen large enough: due to the division processes, we expect that the support of the solution remains essentially on a bounded interval, and the cut-off should not perturb too much the solution. In what follows, the size step *h* = *z_i_*_+1_ -*z_i_* is assumed to be constant. The discrete unknowns *N_i_*, with *i* ∈ { 1, … ,*I* } and *h* = *R/I*, are intended to approximate *N*( *z*_*i*_ ) where *z_i_* = *ih*. The integral that defines the gain term of the division operator is approximated by a simple quadrature rule. For the operator (2) the kernel involves Dirac masses which can be approached by peaked Gaussian. We introduce the operator $T_{\text{Λ}}^{h}:{\mathbb{R}}^{I}\to {\mathbb{R}}^{I}$ defined by


(18) $$\{\begin{array}{l} {\left(T_{\text{Λ}}^{h}N\right)}_{i}=F_{i}-F_{i-1}+h\left(\text{Λ}+a_{i}\right)N_{i}\\ -h^{2}\sum_{j=i}^{I}a\left(z_{j}\right)k(z_{i}|z_{j})N_{j},\\ N_{1}=0 \end{array}$$

where *F*_*i*_ =*V*_*i*+1/2_ *N*_*i*_ represents the convective numerical flux on the grid point *z*_*i*+1/2_ =( *i*+1/2 )*h* , *i* ∈ { 1, … ,*I* } . This definition takes into account that the growth rate is non negative, and applies the upwinding principles. Note that the step size *h* should be small enough to capture the division of small cells, if any. The following statement provides the *a priori* estimate which allows us to determine the shift for the discrete problem.

Theorem 1.1. We suppose that

- *z*↦*V*( *z* ) is a continuous function which lies in *L* ^*∞*^ and it is bounded from below by a positive constant,
-
$h\sum_{j=1}^{I}a\left(z_{j}\right)k\left(z_{i}|z_{j}\right)$ remains bounded uniformly with respect to *h*,
- for any *i*∈{ 1,...,*I*−1 } , there exists *j*∈{ 1,...,*I*−1 } such that *a*(*z*_*j*_)*k*( *z*_*i*_ ∣*z*_*j*_ )>0,
- there exists *Z*_0_ ∈( 0,*∞* ) such that, setting $\bar{N}\left(z\right)=h\sum_{j=2}^{I}k(z_{j}|z)$ , we have $a\left(z\right)\left(\bar{N}\left(z\right)-1\right)\geq \nu_{0}>0$ for any *z* ≥ *Z*_0_.

Let


(23) $$\begin{array}{l} \text{Λ}>\frac{∥V{∥}_{L^{\infty }}}{\underset{j\in \left\{1,\text{...},I\right\}}{\min}|V_{j+1/2}|}\underset{k\in \left\{1,\text{...},I\right\}}{\max}(h\sum_{j=k}^{I}a_{j}k(z_{k}|z_{j}))\\ -\underset{j\in \left\{1,\text{...},I\right\}}{\min}|a_{j}|, \end{array}$$

*and we suppose that R > Z_0_ is large enough. Then*, $T_{\Lambda }^{h}$ *is invertible and there exists a pair μ*>0, *N*∈*ℝ* ^*I*^ *with positive components, such that* $Ker(\left(T_{\Lambda }^{h}{)}^{-1}-\mu \right)=Span\left\{N\right\}$ *. Moreover* $\lambda =\Lambda -\frac{1}{\mu }>0$ .

Note that the sum that defines $\bar{N}\left(z\right)$ is actually reduced over the indices such that *jh*≤*z* ; this quantity is interpreted as the expected number of cells produced from the division of a cell with size *z* so that the forth assumption is quite natural.

*Proof.* Let *f*∈*ℝ* ^*I*^. We consider the equation


$$T_{\text{Λ}}^{h}N=f.$$

We denote $N=S_{\text{Λ}}^{h}f$ the solution. We are going to show that $S_{\text{Λ}}^{h}$ is well defined and satisfies the assumptions of the Perron-Frobenius theorem, see e. g. (47, Theorem 1.37 & Corollary 1.39) or (86, Chapter 5).

It is convenient to introduce the change of unknown *U*_*i*_ =*N*_*i*_ *V*_*i*+1/2_ , ∀*i*∈{ 1,⋯,*I* } . The problem recasts as


(20) $$\{\begin{array}{l} {\left({\tilde{T}}_{\text{Λ}}^{h}U\right)}_{i}=h\frac{f_{i}}{V_{i+1/2}},\text{with}\\ {\left({\tilde{T}}_{\text{Λ}}^{h}U\right)}_{i}=U_{i}-U_{i-1}+h\frac{\Lambda +a_{i}}{V_{i+1/2}}U_{i}\\ \;-h^{2}\sum_{j=i}^{I}\frac{a_{j}}{V_{j+1/2}}k(z_{i}|z_{j})U_{j},\\ U_{1}=0. \end{array}$$

The solution is interpreted as the fixed point of the mapping


$$\xi \mapsto U=A^{h}\xi$$

where *U* is given by *U*_1 = 0_ and


$$U_{i}=U_{i-1}+h^{2}\sum_{j=i}^{I}\frac{a_{j}}{V_{j+1/2}}k\left(z_{i}|z_{j}\right)\xi_{j}+h\frac{f_{i}}{V_{i+1/2}}.$$

We are going to show that *A^h^* is a contraction: ∥*A* ^*h*^ *ξ*∥_*ℓ* ^*∞*^_ ≤*k*∥*ξ*∥_*ℓ* ^*∞*^_ for some *k* < 1. Multiplying (20) by sign (*U_i_*), we obtain


$$\begin{array}{l} \left(1+h\frac{\Lambda +a_{i}}{V_{i}}\right)\text{sign}\left(U_{i}\right)U_{i}=\left(1+h\frac{\Lambda +a_{i}}{V_{i}}\right)\left|U_{i}\right|\\ =\text{sign}\left(U_{i}\right)U_{i-1}+h^{2}\sum_{j=1}^{I}\frac{a_{j}}{V_{j+1/2}}k\left(z_{i}|z_{j}\right)\text{sign}\left(U_{i}\right)\xi_{j}\\ \leq \left|U_{i-1}\right|+h^{2}\sum_{j=i}^{I}\frac{a_{j}}{V_{j+1/2}}k(z_{i}|z_{j})\left|\xi_{j}\right|. \end{array}$$

We multiply this by the weight $\prod_{l=1}^{i-1}\left[1+h\frac{\Lambda +a_{l}}{V_{l+1/2}}\right]$ , where all factors are ≥1 . We get


$$\begin{array}{l} \left|U_{i}\right|\prod_{l=1}^{i}\left[1+h\frac{\Lambda +a_{l}}{V_{l+1/2}}\right]\\ \;\leq \left|U_{i-1}\right|\prod_{l=1}^{i-1}\left[1+h\frac{\Lambda +a_{l}}{V_{l+1/2}}\right]\\ \;+h^{2}\prod_{l=1}^{i}\left[1+h\frac{\Lambda +a_{l}}{V_{l+1/2}}\right]\sum_{j=i}^{I}\frac{a_{j}}{V_{j+1/2}}k(z_{i}|z_{j})\left|\xi_{j}\right|. \end{array}$$

Then, summing over *i*∈{ 2, ... ,*m* } yields


$$\begin{array}{l} \left|U_{m}\right|\prod_{l=1}^{m}\left[1+h\frac{\Lambda +a_{l}}{V_{l+1/2}}\right]\\ \;\leq \left|U_{1}\right|\left[1+h\frac{\Lambda +a_{1}}{V_{3/2}}\right]\\ \;\;+h^{2}\sum_{i=2}^{m}\prod_{l=1}^{i}\left[1+h\frac{\Lambda +a_{l}}{V_{l+1/2}}\right]\sum_{j=i}^{I}\frac{a_{j}}{V_{j+1/2}}k(z_{i}|z_{j})\left|\xi_{j}\right| \end{array}$$

where actually *U*_1_ = 0. It follows that


$$\begin{array}{l} \left|U_{m}\right|\leq h^{2}\sum_{i=2}^{m}\prod_{l=i}^{m}{\left[1+h\frac{\Lambda +a_{l}}{V_{l+1/2}}\right]}^{-1}\sum_{j=i}^{I}\frac{a_{j}}{V_{j+1/2}}k\left(z_{i}|z_{j}\right)\left|\xi_{j}\right|\\ \leq \frac{h^{2}∥\xi {∥}_{\ell^{\infty }}}{\underset{j\in \left\{1,\text{...},I\right\}}{\min}V_{j+1/2}}\sum_{i=2}^{m}\prod_{l=i}^{m}{\left[1+h\frac{\Lambda +a_{l}}{V_{l+1/2}}\right]}^{-1}\sum_{j=i}^{I}a_{j}k\left(z_{i}|z_{j}\right)\\ \leq \frac{h^{2}∥\xi {∥}_{\ell^{\infty }}}{\underset{j\in \left\{1,\text{...},I\right\}}{\min}V_{j+1/2}}∥\sum_{j=i}^{I}a_{j}k\left(z_{i}|z_{j}\right){∥}_{\ell^{\infty }}\\ \sum_{i=2}^{m}{\left[1+h\frac{\Lambda +\underset{l\in \left\{1,\text{...},I\right\}}{\min}a_{l}}{∥V{∥}_{L^{\infty }}}\right]}^{i-m+1}\\ \leq \frac{h∥\xi {∥}_{\ell^{\infty }}}{\underset{j\in \left\{1,\text{...},I\right\}}{\min}V_{j+1/2}}∥\sum_{j=i}^{I}a_{j}k\left(z_{i}|z_{j}\right){∥}_{\ell_{\infty }}\\ {\left[\frac{\Lambda +\underset{l\in \left\{1,\text{...},I\right\}}{\min}a_{l}}{∥V{∥}_{L^{\infty }}}\right]}^{-1} \end{array}$$

Therefore, *A^h^* is a contraction provided (19) holds. This estimate is similar to the condition obtained for the continuous problem, see (32, Proof of Theorem 2, Appendix B); the discretization does not introduce further constraints.

We are now going to show that $T_{\Lambda }^{h}$ is a *M*-matrix when (19) holds. Let *f* ∈ *ℝ* ^*I*^ ∖{ 0 } with non negative components. Let *U* ∈ *ℝ* ^*I*^ satisfy ${\left({\tilde{T}}_{\Lambda }^{h}U\right)}_{i}=h\frac{f_{i}}{V_{i+1/2}}$ . Let *i*_0_ be the index such that *U*_*i*_0__ =min { *U*_*i*_ , *i*∈{ 2, ..., *I* } }. We have


(21) $$\begin{array}{l} U_{i_{0}}\left(1+h\frac{\Lambda +a_{i_{0}}}{V_{i_{0}+1/2}}\right)\\ =U_{i_{0}-1}+h^{2}\sum_{j=i_{0}}^{I}\frac{a_{j}}{V_{j+1/2}}k\left(z_{i_{0}}|z_{j}\right)U_{j}+h\frac{f_{i_{0}}}{V_{i_{0}+1/2}}\\ \geq U_{i_{0}}\left(1+h^{2}\sum_{j=i_{0}}^{I}\frac{a_{j}}{V_{j+1/2}}k\left(z_{i_{0}}|z_{j}\right)\right)+h\frac{f_{i_{0}}}{V_{i_{0}+1/2}} \end{array}$$

Since *f*_*i*_0__ ≥0 , we get


$$U_{i_{0}}\underset{>0\text{by}\left(19\right)}{\underbrace{\left(\frac{\Lambda +a_{i_{0}}}{V_{i_{0}+1/2}}-h\sum_{j=i_{0}}^{I}\frac{a_{j}}{V_{j+1/2}}k\left(z_{i_{0}}|z_{j}\right)\right)}}\geq 0$$

which tells us that *U*_*i*_0__ ≥0. Suppose *U*_*i*_0__ =0 for some *i*_0_ >1. Coming back to (21), we deduce that *U*_*i*_0_ −1_ vanishes too, and so on and so forth, we obtain *U*_1_ =⋯*U*_*i*_0__ =0 . Finally, we use the irreductibility assumption iii): we can find *j*_0_ > *i*_0_ such that $\frac{a_{j_{0}}}{V_{j_{0}+1/2}}k\left(z_{i_{0}}|z_{j_{0}}\right)>0$ and (21) implies $\frac{a_{j_{0}}}{V_{j_{0}+1/2}}k\left(z_{i_{0}}|z_{j_{0}}\right)U_{j_{0}}=0$ , so that *U*_*j*_0__ =0. We deduce that *U* = 0, which contradicts *f*≠0 . Therefore the components of *U* are positive, but *U*_1_.

We conclude by applying the Perron-Froebenius theorem to ${\left(T_{\Lambda }^{h}\right)}^{-1}$ , (86). It remains to prove that $\lambda =\text{Λ}-\frac{1}{\mu }$ is positive, with *μ* the spectral radius of ${\left(T_{\Lambda }^{h}\right)}^{-1}$ . To this end, we make use of assumption iv). We set *Z*_0_ = *i*_0_ *h*. We argue by contradiction, supposing that *λ*=Λ−1/*μ*<0. We consider the eigenvector with positive components and normalized by the condition $h\sum_{i=1}^{I}U_{i}=1$ . We have


$$\begin{array}{l} ({\tilde{T}}_{0}^{h}U)i=U_{i}-U_{i-1}+\frac{a_{i}}{V_{i+1/2}}hU_{i}\\ -h^{2}\sum_{j=}^{I}\frac{a_{j}}{V_{j+1/2}}k\left(z_{i}|z_{j}\right)U_{j}=-\lambda U_{i}\geq 0 \end{array}$$

It follows that, for *m*≥*i*_0_ ,


$$\begin{array}{c} U_{m}\geq -h\sum_{i=2}^{m}\frac{a_{i}}{V_{i+1/2}}U_{i}+-h^{2}\sum_{i=2}^{m}\sum_{j=i}^{I}\frac{a_{j}}{V_{j+1/2}}k(z_{i}|z_{j})U_{j}\\ \geq -h\sum_{i=2}^{m}\frac{a_{i}}{V_{i+1/2}}U_{i}+h\sum_{j=2}^{m}\left(h\sum_{i=2}^{j}k(z_{i}|z_{j})\right)\frac{a_{j}}{V_{j+1/2}}U_{j}\\ \geq -h\sum_{i=2}^{m}\frac{a_{i}}{V_{i+1/2}}U_{i}+h\sum_{j=2}^{m}\bar{N}\left(z_{j}\right)\frac{a_{j}}{V_{j+1/2}}U_{j}\\ \geq h\sum_{i=2}^{m}\left(\bar{N}\left(z_{i}\right)-1\right)\frac{a_{i}}{V_{i+1/2}}U_{i}\\ \geq h\sum_{i=i_{0}}^{m}\left(\bar{N}\left(z_{i}\right)-1\right)\frac{a_{i}}{V_{i+1/2}}U_{i}\geq \frac{\nu_{0}}{∥V{∥}_{L^{\infty }}}h\sum_{i=i_{0}}^{m}U_{i}. \end{array}$$

It implies


$$1=h\sum_{m=1}^{I}U_{m}\geq h\sum_{m=i_{0}}^{I}U_{m}\geq h\left(I-i_{0}\right)\frac{\nu_{0}}{∥V{∥}_{L^{\infty }}}h\sum_{i=i_{0}}^{m}U_{i}.$$

We arrive at


$$1\geq \left(R-Z_{0}\right)\frac{\nu_{0}}{∥V{∥}_{L^{\infty }}},$$

a contradiction when *R* is chosen large enough (but how large *R* should be does not depend on *h*). Therefore, we conclude that λ > 0.

### 6.2 Numerical Approximation of (λ, N)

We compute (an approximation of) the eigenpair (λ, *N*) by using the inverse power method which finds the eigenvalue of ${\left(T_{\Lambda }^{h}\right)}^{-1}$ with largest modulus:

• We pick Λ verifying (19).

• We compute once for all the *LU* decomposition of the matrix $T_{\Lambda }^{h}$ .

• We choose a threshold 0 < *ε* ≪ 1.

• We start from a random vector *N* ^(0)^ and we construct the iterations


$$LUq^{\left(k+1\right)}=N^{\left(k\right)}$$

$$N^{\left(k+1\right)}=\frac{q^{\left(k+1\right)}}{∥q^{\left(k+1\right)}∥}$$

until the relative error $\frac{∥N^{\left(k+1\right)}-N^{\left(k\right)}∥}{∥N^{\left(k\right)}∥}\leq \varepsilon$ is small enough. Then, given the last iterate *N* ^(^ *^K^* ^)^, we set $LUq=N^{\left(K\right)},\tilde{\mu }=\frac{q\cdot N^{\left(K\right)}}{N^{\left(K\right)}\cdot N^{\left(K\right)}},\text{ and }\tilde{\lambda }=\text{Λ}-1/\tilde{\mu }.$

This approach relies on the ability to approximate correctly the eigenpair of the growth-fragmentation operator. In particular, it is important to preserve the algebraic multiplicity. This issue is quite subtle and it is known that the pointwise convergence of the operator is not enough to guarantee the convergence of the eigenelements and the consistency of the invariant subspaces, see (48) for relevant examples. This question has been thoroughly investigated in (48, 49) which introduced a suitable notion of stability. It turns out that one needs a uniform convergence of the operators. Namely, here, we should check that $∥{\left(T_{\text{Λ}}^{I}\right)}^{-1}-{\left(T_{\text{Λ}}\right)}^{-1}∥\to 0$ as *I*→*∞*. In the present framework, a difficulty relies on the fact that the size variable lies in an unbounded domain, which prevents for using usual compactness arguments. For this reason, we introduce a truncated version of the problem, which has also to be suitably regularized. Let us denote by $T_{\Lambda }^{R,\varepsilon }$ the corresponding operator, where *ε* represents the regularization parameter. This truncated and regularized perator appeared already in (32). Indeed, we know from (32) that $∥T_{\text{Λ}}^{R,\varepsilon }-T_{\text{Λ}}∥\to 0$ as *R*→*∞* and ε→0, hence, this implies that $∥{\left(T_{\text{Λ}}^{R,\varepsilon }\right)}^{-1}-{\left(T_{\text{Λ}}\right)}^{-1}∥\to 0$ as *R*→*∞* and ε→0 by continuity of the map Π:*T*_Λ_ ↦ ( *T*_Λ_ )^−1^ . Moreover, ${\left(T_{\text{Λ}}^{R,\varepsilon }\right)}^{-1}$ is well-defined, continuous and compact, see (32, Appendix. B). The discrete operators ${\left(T_{\text{Λ}}^{I}\right)}^{-1}$ converge pointwise to ${\left(T_{\text{Λ}}^{R,\varepsilon }\right)}^{-1}$ , and the compactness of ${\left(T_{\text{Λ}}^{R,\varepsilon }\right)}^{-1}$ ensures that the discrete operator converges uniformly to ${\left(T_{\text{Λ}}^{R,\varepsilon }\right)}^{-1}$ , for 0<*R*<ε and 0<ε<1 fixed (see (49) for more details on this fact). Following (49), we deduce that the numerical eigenelements (*λ^I^*, *N^I^*) converges to ( *λ* ^*R*,ε^ , *N* ^*R*,ε^ ), the eigenelements of ${\left(T_{\text{Λ}}^{R,\varepsilon }\right)}^{-1}$ , while preserving their algebraic multiplicity. Finally the uniform convergence $∥{\left(T_{\text{Λ}}^{R,\varepsilon }\right)}^{-1}-{\left(T_{\text{Λ}}\right)}^{-1}∥\to 0$ as *R*→*∞* and ∈→0 ensures the convergence of ( *λ* ^*R*,ε^ , *N* ^*R*,ε^ ), to (*λ*, *N*) (32).

### 6.3 Numerical Results

For some specific fragmentation kernels and growth rates, the eigenpair $\left(\lambda ,\bar{N}\right)$ is explicitly known, see (32). We can use these formula to check that the algorithm is able to find the expected values and profiles. To this end, we introduce the relative errors


$$E_{\lambda }^{h}=\frac{\left|\lambda -\tilde{\lambda }\right|}{\tilde{\lambda }}\;\text{ and }E_{V}^{h}=h\sum_{i=1}^{I}\left|N_{i}^{\left(K\right)}-N\left(ih\right)\right|$$

where *N* ^(^ *^K^* ^)^ and *N* are both normalized by $h\sum_{i=1}^{I}N_{i}^{\left(K\right)}=h\sum_{i=1}^{I}N\left(ih\right)=1$ .

**Mitosis fragmentation kernel**. We start with the binary division kernel:


(22) $$k\left(z|z^{\prime }\right)=\delta_{z^{\prime }=2z}$$

The associated division operator is described by (2). We assume that *a* and *V* are constant. In this specific case the eigenpair is given by


(23) $$\begin{array}{cc} \lambda =a, & N\left(z\right)=N\sum_{n=0}^{\infty }{\left(-1\right)}^{n}\alpha_{n}\text{exp }\left(-2^{n+1}\frac{a}{V}z\right), \end{array}$$

with *N* > 0 an appropriate normalizing constant and ( *α*_*n*_ )_*n*∈*ℕ*_ is the sequence defined by the recursion


$$\begin{array}{cc} \alpha_{0}=1, & \alpha_{n}=\frac{2}{2^{n}-1}\alpha_{n-1.} \end{array}$$

In practice we shall use a truncated version of the series that defines *N*. For the numerical tests, we use the parameters collected in **Table 4**.

**Table 4:** Data for the numerical tests: binary division kernel.

*a*	*V*	*R*	*∈*
4	0.6	5	10^-6^

**Figure 10:**
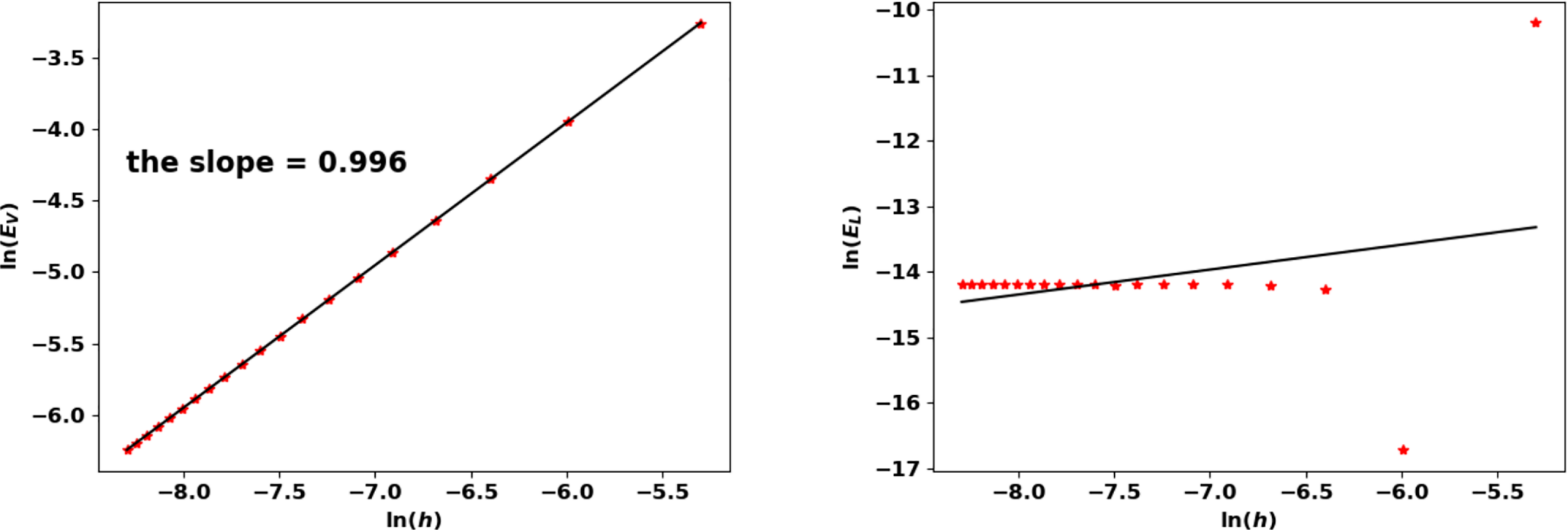
Binary division kernel: convergence rates of (*λ* ^(^ *^K^* ^)^, *N* ^(^ *^K^* ^)^) with respect to *h*.

With this threshold *ε*, the approached eigenpair is reached in 43 iterations, independently of the size step. **Figure 10** represents the evolution of the error $E_{V}^{h}$ as a function of *h* in a log-log scale, see also **Table 5**: *N* ^(^ *^K^* ^)^ approaches *N* at order 1. The rate improves when using a quadrature rule with a better accuracy. For this test, the approximation of the eigenvalue is already accurate with a coarse grid; it is simply driven by the threshold *ε* and $E_{L}^{h}$ does not significantly change with *h*.

**Table 5:** Binary division kernel: errors for several number of grid points.

Number of cells	*E_λ_*	*E_V_*
1000	3.73 × 10^-5^	3.83 × 10^-2^
2000	5.68 × 10^-8^	1.93 × 10^-2^
4000	6.77 × 10^-7^	9.69 × 10^-3^
8000	6.84 × 10^-7^	4.85 × 10^-3^

**Uniform fragmentation**. The Uniform fragmentation kernel is defined by:


$$k(z|z^{\prime })=\frac{1}{z^{\prime }}1_{0\leq z\leq z^{\prime }}.$$

We apply the algorithm for the following two cases:

*V*(*z*) = *V*_0_ and *a*(*z*) = *a*_0_ *z*. We have $\lambda =\sqrt{a_{0}V_{0}}$ and


$$N\left(z\right)=2\sqrt{\frac{a_{0}}{V_{0}}}\left(Z+\frac{Z^{2}}{2}\right)\text{exp }\left(-Z-\frac{Z^{2}}{2}\right).$$

**Figure 11:**
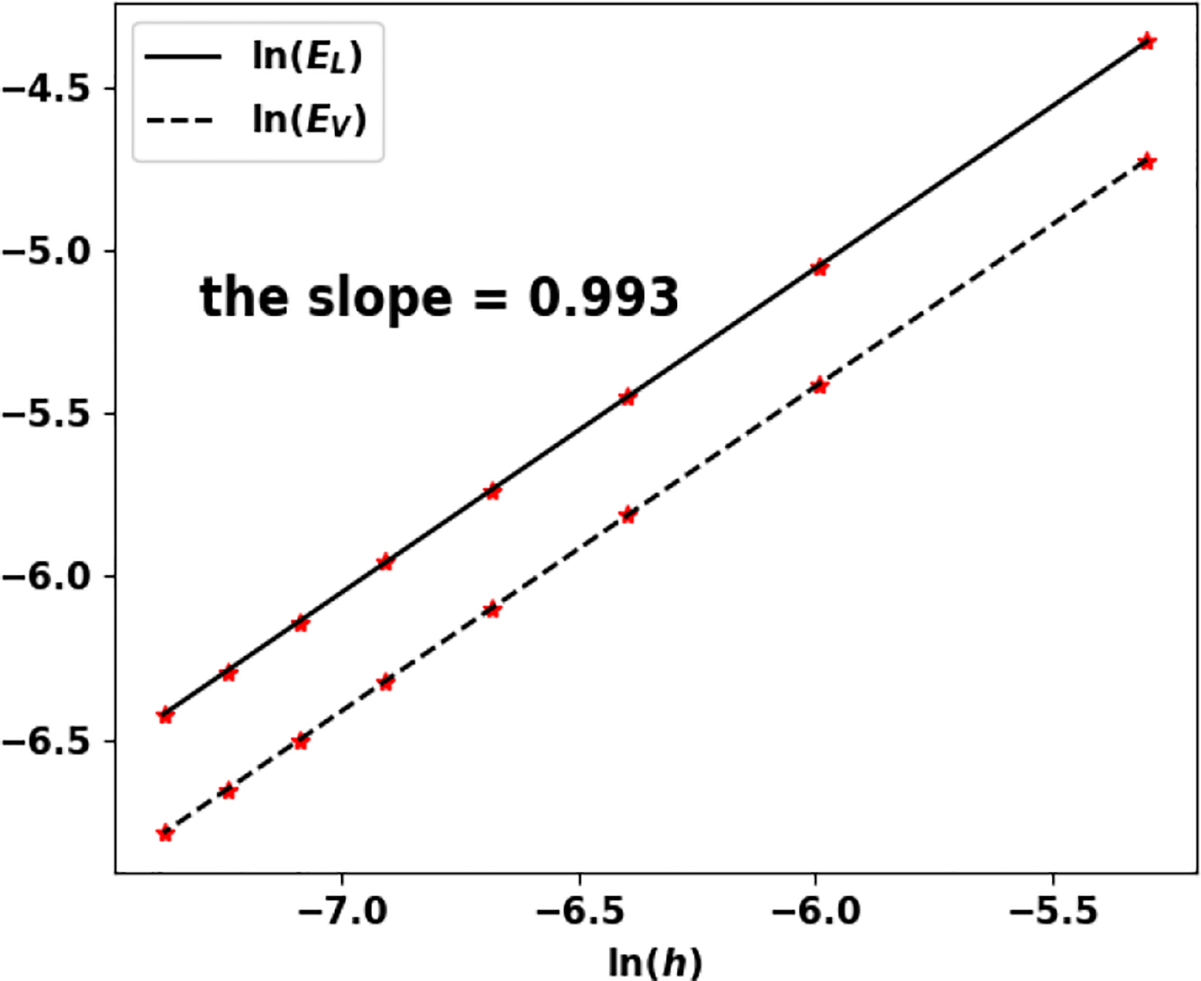
Uniform fragmentation, ex. 1: rate of convergence to the exact eigenpair with respect to *h*.

We still use the values in **Table 4** (especially, *a*_0_ = *a* and *V*_0_ = *V*). The approximated eigenpair is obtained in 84 iterations and, as in the previous test, it does not change with the size step. In this case, both the eigenvalue and the eigenfunction are approached at order 1, see **Table 6** and **Figure 11**.

**Table 6:** Uniform fragmentation, ex. 1: errors for several number of grid points.

Number of cells	*E_λ_*	*E_V_*
1000	1.30 × 10^-2^	8.89 × 10^-3^
2000	6.43 × 10^-3^	4.50 × 10^-3^
4000	3.23 × 10^-3^	2.24 × 10^-3^
8000	1.62 × 10^-3^	1.13 × 10^-3^

*V*(*z*) = *V*_0_ *z* and *a*(*z*) = *a*_0_ *z^n^* with *n*∈*ℕ*∖{ 0 } . The eigenpair is defined by the following formula:


$$\begin{array}{ccc} n=1 & \lambda =V_{0} & N\left(z\right)=\frac{a_{0}}{V_{0}}\exp\;\left(-\frac{a_{0}}{V_{0}}z\right)\\ n=2 & \lambda =V_{0} & N\left(z\right)=\frac{2a_{0}}{\pi V_{0}}\exp\;\left(-\frac{a_{0}}{2V_{0}}z^{2}\right)\\ n & \lambda =V_{0} & N\left(z\right)={\left(\frac{a_{0}}{nV_{0}}\right)}^{\frac{1}{n}}\frac{n}{\Gamma \left(\frac{1}{n}\right)}\exp\;\left(-\frac{a_{0}}{nV_{0}}z^{n}\right) \end{array}$$

**Figure 12:**
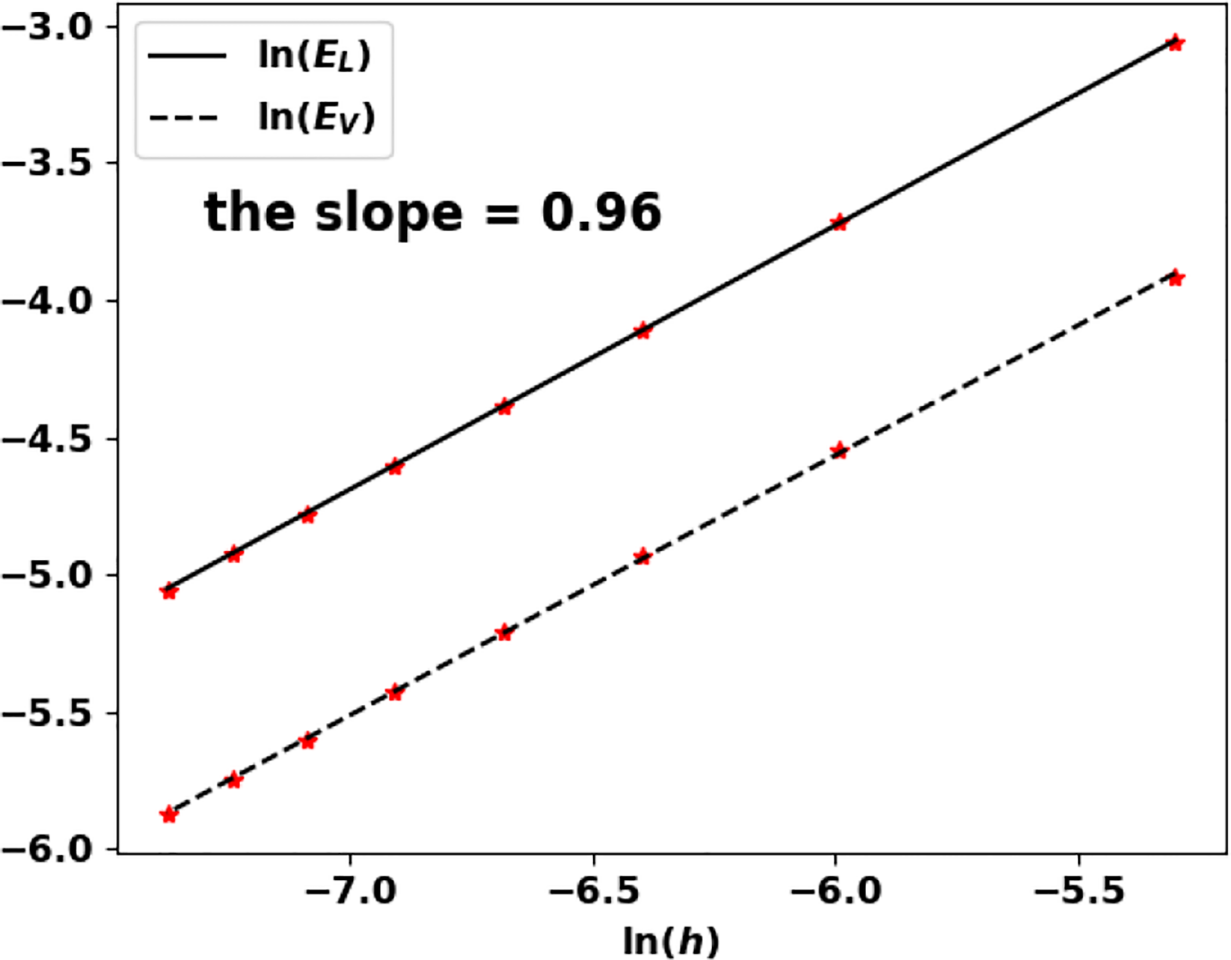
Uniform fragmentation, ex. 2 case *n* = 1: rate of convergence to the exact eigenpair with respect to *h*.

**Figure 13:**
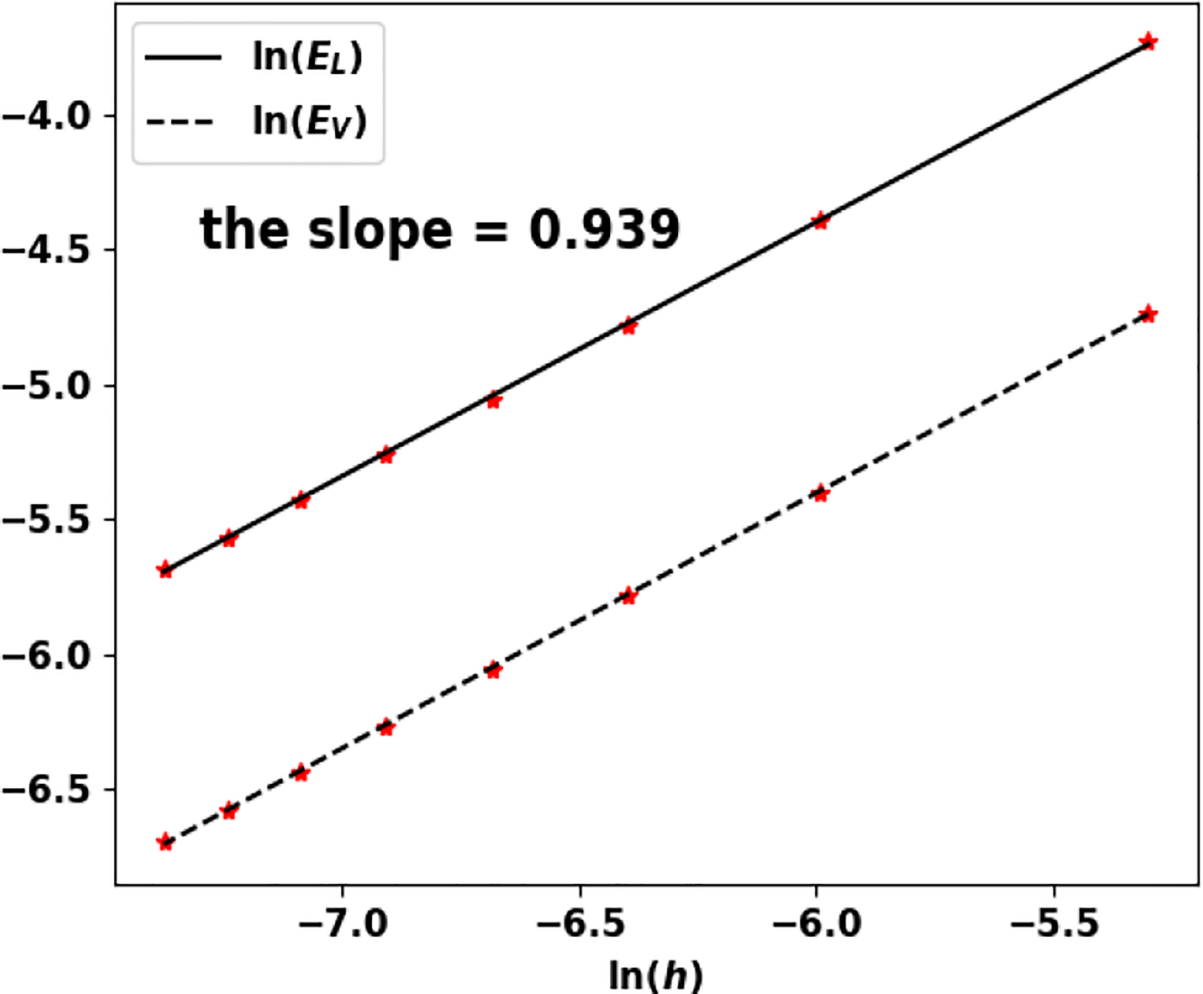
Uniform fragmentation, ex. 2: rate of convergence to the exact eigenpair with respect to *h*.

Note that the growth rate *V* vanishes and Theorem 1.1 does not apply as such. Nonetheless, the algorithm works well and still captures the eigenpair. We perform the test for *n* = 1 and *n* = 2 and the results are recorded in **Table 7**; **Figure 12** and **Table 8**; **Figure 13**, respectively.

**Table 7:** Uniform fragmentation, ex. 2, case *n* = 1: errors for different number of cells.

Number of cells	*E_λ_*	*E_V_*
1000	4.70 × 10^-2^	2 × 10^-2^
2000	2.43 × 10^-2^	1.06 × 10^-2^
4000	1.25 × 10^-2^	5.5 × 10^-3^
8000	6.39 × 10^-3^	2.81 × 10^-3^

**Table 8:** Uniform fragmentation, ex. 2, case *n* = 2: errors for different number of cells.

Number of cells	*E_λ_*	*E_V_*
1000	2.39 × 10^-2^	8.81 × 10^-2^
2000	1.23 × 10^-3^	4.53 × 10^-3^
4000	6.41 × 10^-3^	2.35 × 10^-3^
8000	3.41 × 10^-3^	1.24 × 10^-3^

## 7 Sensitivity Analysis on the Equilibrium Mass

Having an efficient procedure to predict the residual mass of the equilibrium phase also opens perspectives to discuss the influence of the parameters. This can provide useful hints for the design and the optimization of anti-tumor therapies. We address this issue by performing a global sensitivity analysis on the immune-controlled tumor mass. Sensitivity analysis also provides information on the quantification of uncertainty in the model output with respect to the uncertainties in the input parameters. We remind the reader that the equilbrium mass is seen as a function of the parameters in **Table 1**:


(24) $$\mu_{1}=f(a,A,p,\chi ,D,\gamma ).$$

We consider that the input parameters are independent random variables uniformly distributed in an interval [ *x*_1_ ,*x*_2_ ]⊂( 0,*∞* ) :


(25) $$M=\left(a,A,p,\chi ,D,\gamma \right)\text{ with }M_{i}∼U\left(x_{1},x_{2}\right).$$

The pillar of the Sobol sensitivity analysis is the decomposition of *f* into 2*^n^* - 1 summands of increasing dimensions:


(26) $$\begin{array}{l} f\left(M\right)=f_{0}+\sum_{i=1}^{n}f_{i}\left(M_{i}\right)\\ +\sum_{1\leq i<j\leq n}f_{ij}\left(M_{i},M_{j}\right)+\cdots +f_{1\cdots n}\left(M_{1},\cdots ,M_{n}\right), \end{array}$$

Where


(27) $$\begin{array}{cc} \frac{1}{x_{2}-x_{1}}\int_{\left[x_{1},x_{2}\right]}f_{i_{1}\cdots i_{p}}\left(M_{i_{1}\cdots i_{p}}\right)\text{ d}M_{i_{k}}=0 & \text{for }k\in \left\{1,\text{...},p\right\}, \end{array}$$

(28) $$f_{0}=\frac{1}{{\left(x_{2}-x_{1}\right)}^{n}}\int_{{\left[x_{1},x_{2}\right]}^{n}}f\left(M\right)\text{ d}M,$$

(29) $$\int_{{\left[x_{1},x_{2}\right]}^{n}}f_{i_{1}\cdots i_{p}}\left(M_{i_{1}\cdots i_{p}}\right)f_{j_{1}\cdots j_{p}}\left(M_{j_{1}\cdots j_{p}}\right)\text{ d}M=0,$$

and *M*_*i*_1_ ⋯*i*_*p*__ =( *M*_*i*_1__ ,⋯*M*_*i*_*p*__ ) . The existence and uniqueness of the above decomposition has been proven in (69), given *f* a square integrable function. Owing to the orthogonality condition (29), the total variance of *f* reads:


(30) $$V=\text{Var}\left(f\left(M\right)\right)=\frac{1}{{\left(x_{2}-x_{1}\right)}^{n}}\int_{{\left[x_{1},x_{2}\right]}^{n}}f{\left(M\right)}^{2}\text{ d}M-f_{0}^{2}.$$

Given (26), *V* can be decomposed as follows:


(31) $$V=\sum_{i=1}^{n}V_{i}+\sum_{1\leq i<j\leq n}V_{ij}+\cdots +V_{1\cdots n},$$

where the terms *V*_*i*_1_ ⋯*i*_*p*__ , called partial variances read:


(32) $$V_{i_{1}\cdots i_{p}}=\frac{1}{{\left(x_{2}-x_{1}\right)}^{n}}\int_{{\left[x_{1},x_{2}\right]}^{n}}f_{i_{1}\cdots i_{p}}^{2}\text{ d}M_{i_{1}}\cdots \text{ d}M_{i_{p}}.$$

Following the description in (69), the Sobol’ sensitivity indices are defined as follows:


(33) $$S_{i_{1}\cdots i_{p}}=\frac{V_{i_{1}\cdots i_{p}}}{V}$$

They verify


(34) $$\sum_{i=1}^{n}S_{i}+\sum_{1\leq i<j\leq n}S_{ij}+\cdots +S_{1\cdots n}=1.$$

Each index *S*_*i*_1_ ⋯*i*_*p*__ measures how the total variance of *f* is affected by uncertainties in the set of input parameters *i*_1_ ⋯*i*_*p*_. An equivalent definition of the above indices is given by [see (68)]:


(35) $$\begin{array}{cc} V_{i}=\text{Var}(E(Y|M_{i})), & V_{ij}=\text{Var}(E(Y|M_{i},M_{j}))-V_{i}-V_{j},\ldots \end{array}$$

The total effect of a specific input parameter *i* is evaluated by the so-called total sensitivity index $S_{T}^{\left(i\right)}$ , the sum of the sensitivity indices which contain *i*:


(36) $$S_{T}^{\left(i\right)}=\sum_{C_{i}}S_{i_{1}\cdots i_{p}}$$

where *C*_*i*_ ={ ( *i*_1_ ⋯*i*_*p*_ ):∃*m*∈{ 1,...,*p* }, *i*_*m*_ =*i* }. In practice, the sensitivity indices that are needed to discriminate the impact of the parameters are the first, second and total Sobol’ sensitivity indices. The above indices are computed using Monte Carlo simulations combined with a careful sampling of the parameters space in order to reduce the computational load and the number of model evaluations. For this purpose, the following estimators can be derived using two different *N* samples *A* and *B*, see (68, 74),


(37) $$\hat{f_{0}}=\frac{1}{N}\sum_{l=1}^{N}f\left(M_{l}\right),$$

(38) $$\hat{V}=\frac{1}{N}\sum_{l=1}^{N}f^{2}\left(M_{l}\right)-{\hat{f}}_{0}^{2},$$

(39) $${\hat{V}}_{i}=\frac{1}{N}\sum_{l=1}^{N}f\left(M_{\left(-i\right)l}^{\left(A\right)},M_{il}^{\left(A\right)}\right)f\left(M_{\left(-i\right)l}^{\left(B\right)},M_{il}^{\left(A\right)}\right)-{\hat{f}}_{0}^{2},$$

(40) $${\hat{V}}_{ij}=\frac{1}{N}\sum_{l=1}^{N}f\left(M_{-\left(i,j\right)l}^{\left(A\right)},M_{il}^{\left(A\right)},M_{jl}^{\left(A\right)}\right)f\left(M_{-\left(i,j\right)l}^{\left(B\right)},M_{il}^{\left(A\right)},M_{jl}^{\left(A\right)}\right)-{\hat{f}}_{0}^{2}-{\hat{V}}_{i}-{\hat{V}}_{j}$$

Here the notation *M*_−( *i*_1_ ⋯*i*_*p*_ )*l*_ stands for the *l*-th sample line where we get rid of the points corresponding to the indices *i*_1_ ,⋯,*i*_*p*_. The total sensitivity (87) is given by:


(41) $$S_{T_{i}}=1-S_{-i}$$

where *S_-i_* is the sum of all the sensitivity indices that do not contain the index *i*. Hence, the total sensitivity index estimator reads:


(42) $${\hat{S}}_{T_{i}}=1-\frac{{\hat{V}}_{-i}}{\hat{V}}$$

Where


$${\hat{V}}_{-i}=\frac{1}{N}\sum_{l=1}^{N}f\left(M_{\left(-i\right)l}^{\left(A\right)},M_{il}^{\left(A\right)}\right)f\left(M_{\left(-i\right)l}^{\left(A\right)},M_{il}^{\left(B\right)}\right)-{\hat{f}}_{0}^{2}.$$

## Data Availability Statement

The datasets presented in this study can be found in online repositories. The names of the repository/repositories and accession number(s) can be found below: https://github.com/atsoukevin93/tumorgrowth.

## Author Contributions

Conception and design: KA, VB, and TG; Development of methodology: KA, VB, and TG; Acquisition of data (provided animals, acquired and managed patients, provided facilities, etc.): FA, VB, and SK; Analysis and interpretation of data (e.g., statistical analysis, biostatistics, computational analysis): KA, VB, and TG; Writing, review, and/or revision of the manuscript: KA, FA, VB, and TG; Study supervision: FA, VB, and TG. All authors contributed to the article and approved the submitted version.

## Funding

This work was supported by the French Government (National Research Agency, ANR) through the “Investments for the Future” programs LABEX SIGNALIFE ANR-11-LABX-0028 and IDEX UCAJedi ANR-15-IDEX-01.

## Conflict of Interest

The authors declare that the research was conducted in the absence of any commercial or financial relationships that could be construed as a potential conflict of interest.

## Publisher’s Note

All claims expressed in this article are solely those of the authors and do not necessarily represent those of their affiliated organizations, or those of the publisher, the editors and the reviewers. Any product that may be evaluated in this article, or claim that may be made by its manufacturer, is not guaranteed or endorsed by the publisher.
